# Adaptive tail-length evolution in deer mice is associated with differential *Hoxd13* expression in early development

**DOI:** 10.1038/s41559-024-02346-3

**Published:** 2024-02-20

**Authors:** Evan P. Kingsley, Emily R. Hager, Jean-Marc Lassance, Kyle M. Turner, Olivia S. Harringmeyer, Christopher Kirby, Beverly I. Neugeboren, Hopi E. Hoekstra

**Affiliations:** 1 Department of Organismic & Evolutionary Biology, Department of Molecular & Cellular Biology, Museum of Comparative Zoology and Howard Hughes Medical Institute, Harvard University, Cambridge, MA USA; 2 Present Address: Department of Genetics, Harvard Medical School, Boston, MA USA; 3 Present Address: Department of Biomedical Engineering, Boston University, Boston, MA USA; 4 Present Address: GIGA Institute, University of Liège, Liège, Belgium; 5 Present Address: Centre for Teaching Support & Innovation, University of Toronto, Toronto, Ontario Canada; 6 Present Address: Environmental Health and Safety, Harvard University, Cambridge, MA USA

**Keywords:** Evolutionary developmental biology, Evolutionary genetics

## Abstract

Variation in the size and number of axial segments underlies much of the diversity in animal body plans. Here we investigate the evolutionary, genetic and developmental mechanisms driving tail-length differences between forest and prairie ecotypes of deer mice (*Peromyscus maniculatus*). We first show that long-tailed forest mice perform better in an arboreal locomotion assay, consistent with tails being important for balance during climbing. We then identify six genomic regions that contribute to differences in tail length, three of which associate with caudal vertebra length and the other three with vertebra number. For all six loci, the forest allele increases tail length, indicative of the cumulative effect of natural selection. Two of the genomic regions associated with variation in vertebra number contain Hox gene clusters. Of those, we find an allele-specific decrease in *Hoxd13* expression in the embryonic tail bud of long-tailed forest mice, consistent with its role in axial elongation. Additionally, we find that forest embryos have more presomitic mesoderm than prairie embryos and that this correlates with an increase in the number of neuromesodermal progenitors, which are modulated by Hox13 paralogues. Together, these results suggest a role for *Hoxd13* in the development of natural variation in adaptive morphology on a microevolutionary timescale.

## Abstract (web summary)

Forest ecotypes of deer mice have longer tails than prairie ecotypes. This study shows that this difference is adaptive and involves changes in six genomic regions, one of which is an allele-specific reduction in *Hoxd13* expression that leads to tail elongation.

## Main

Understanding the genetic and developmental bases of evolutionary changes in morphology, especially those that affect fitness in the wild, is a key goal of modern biology^1–4^. A major source of morphological change on a macroevolutionary scale in animals is the alteration in the numbers and identities of serially homologous body parts along the anterior–posterior axis—from body segments of arthropods and annelids to vertebrae in the spinal column of vertebrates. Much work has been done to understand the mechanistic basis of changes in segment identity, for example, how shifts in the expression profiles of developmental genes are associated with large-scale changes in the body plan of invertebrates^5,6^ and with transposition of vertebral identities in vertebrates^7^. However, relatively little is known about how naturally occurring genetic changes act through developmental processes to produce differences in segment size and/or number that occur in the wild, and whether the same mechanisms involved in macroevolutionary changes contribute to variation within or between closely related species.

In vertebrates, segment identity and number are determined embryonically. During the process of main body axis segmentation, the embryonic segments—somites—form rhythmically from anterior to posterior as the embryo elongates. As somite formation proceeds, the unsegmented presomitic mesoderm (PSM) shrinks and segmentation ends when somite formation catches up to the tip of the elongating tail^8–10^. Periodic expression of notch pathway components regulates the rate of segment formation^11–13^, and posterior axis elongation is promoted by Wnt and fibroblast growth factor activity in the tail bud^14^. Changes to the dynamics of somite formation and/or posterior elongation are thought to largely underlie evolutionary differences in segment number^9^. Concomitantly, regionalized morphologies of axial segments are influenced by expression domains of Hox genes, the boundaries of which correlate to regional vertebral identity^7,15–17^. The role of Hox genes in conferring segmental identity are complemented by their role in regulating axial elongation. In particular, activation of posterior Hox genes correlates with a slowdown of axis elongation via repression of Wnt activity^18–20^.

In vertebrates, one of the most variable segmental morphologies is vertebra number, especially those in the tail. In mammals, the number of cervical vertebrae is nearly uniform: the vast majority of mammals have seven cervical vertebrae with a few well-known exceptions^21–23^. In contrast, the caudal region is the most evolutionarily labile region of the vertebral column, ranging from as few as three vertebrae in the coccyx of great apes to more than 45 in the long-tailed pangolin^23,24^. Tail morphology is often closely associated with its function—from propulsion during swimming^25^, a counterweight during bipedal saltation^26^ or as a rudder during gliding^27^ or powered flight^28^—suggesting a role for natural selection in the evolution of the tail.

The deer mouse (*Peromyscus maniculatus*) occupies diverse habitats across its extensive range in North America and shows striking variation in several morphological traits, most notably, tail length^29–31^. At the extreme, deer mice occupying forest habitat can have tails that are 60% longer (approximately 45 mm difference) than those occupying prairie habitat^32^. Remarkably, this morphological divergence between the forest and prairie ecotypes evolved recently, probably as a result of the northward retreat of glaciers approximately 10,000 years ago that opened up new forest habitats, which prairie mice could colonize and where selection may have favoured the evolution of long tails^29,32,33^. Indeed, in this species, long tails may be beneficial for arboreal locomotion: long tails have evolved multiple times independently in forested habitat^32,33^; tail amputation adversely affects climbing performance, disproportionately reducing performance in forest mice^34^; and specifically, longer tails are predicted to more effectively promote balance than short tails^35^.

**Fig. 1:**
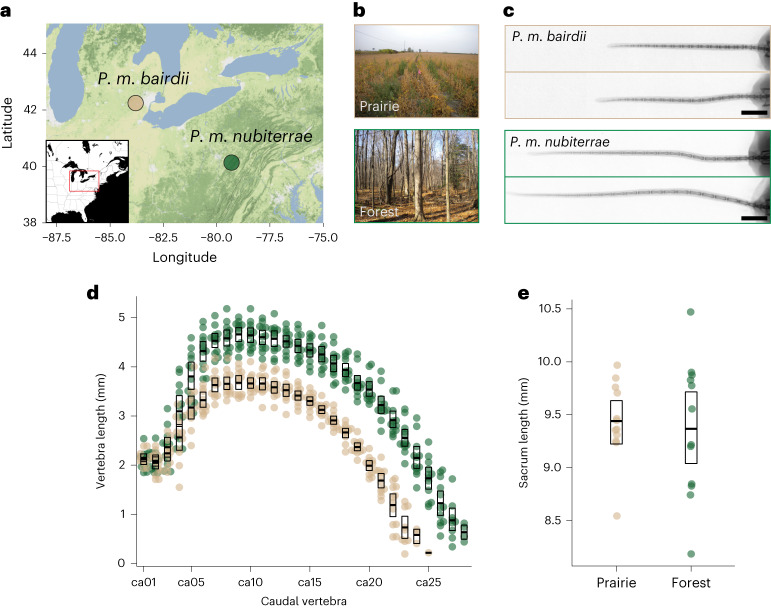
Source populations and morphological traits of wild-caught, laboratory-reared deer mice. **a**, A terrain map showing the trapping locations of mice used in this study from southern Michigan (prairie) and northwestern Pennsylvania (forest). Prairie ecotype (*P.* *m.* *bairdii*, tan), forest ecotype (*P.* *m.* *nubiterrae*, green). Map tiles by Stamen Design, under CC BY 3.0. Data by OpenStreetMap, under ODbL. **b**, Photographs represent typical habitat of each ecotype. **c**, Representative radiographs of lab-born prairie (top, *n* = 2) and forest (bottom, *n* = 2) mouse tails show differences in tail length. Scale bar, 10 mm. **d**, A scatter plot of caudal vertebra lengths shows that both length and number of caudal vertebrae contribute to differences in tail length between prairie (*n* = 12, tan) and forest (*n* = 12, green) mice. **e**, Plots showing that sacral vertebra length, a proxy for body size, does not differ between ecotypes. The boxes show means and bootstrapped 95% confidence limits of the mean.

In this Article, we investigate the potential behavioural consequences and the genetic and developmental causes for natural differences in tail length by comparing two representatives of classic deer mouse ecotypes—*P.* *m.* *nubiterrae* (forest) and *P.* *m.* *bairdii* (prairie)^29^—found in eastern North America (Fig. 1a,b). First, we show that these two subspecies differ dramatically in their climbing performance, in the direction expected on the basis of their tail length differences. Then, using a forward-genetics approach, we identify regions of the genome harbouring mutations that affect tail length. We link changes in expression of a gene in one of these regions, *Hoxd13*, to differences in PSM size and its neuromesodermal progenitors (NMPs) as a likely developmental mechanism underlying vertebra number differences. Together, these data suggest a role for Hox genes in microevolutionary changes underlying natural variation in morphology.

## Results

### Tail-length difference is due to vertebral length and number

To characterize the difference in tail length between ecotypes, we measured total tail length, caudal vertebra lengths and caudal vertebra number from x-ray images of laboratory-raised forest and prairie mice (*n* = 12 for each ecotype; Fig. 1c and Supplementary Fig. 1). We found that forest mice have tails that are 1.4 times longer than those of prairie mice (forest, mean tail length: 84.5 mm (s.d. 7.07 mm) and prairie: 60.2 mm (s.d. 3.51 mm)), which largely recapitulates the difference observed in wild-caught specimens (1.5-fold difference^32^). As this difference was maintained when mice were raised in a common environment, variation in tail length probably has a strong genetic (that is, inherited) component. Specifically, we estimated that genetic variants segregating between ecotypes could explain as much as 88% of the total variance in tail length, based on the distribution of tail lengths in mice from our laboratory colonies.

The difference in overall tail length was due to a difference in both length of caudal vertebrae and number of vertebrae. As the lengths of vertebrae along the tail of an individual were highly correlated (mean correlation between neighbouring vertebrae of 0.84; Supplementary Fig. 2), hereafter we focus on the length of the longest vertebra. We found that forest mice have longer caudal vertebrae than prairie mice: the mean length of the longest forest vertebra was significantly longer than that in prairie mice (1.23 times longer; *t*-test, *t* = −4.3, d.f. 7.4 and *P* = 2 × 10^−3^; forest, 4.73 mm (s.d. 0.27 mm) and prairie, 3.75 mm (s.d. 0.23 mm)). In fact, nearly half of the vertebrae in the forest tail (12 positions, ca6–ca18) are longer, on average, than any vertebra in the prairie tail (Fig. 1d). By contrast, we did not find length differences between ecotypes in vertebrae from other more cranial regions (for example, sacral vertebrae; Fig. 1e and Supplementary Fig. 1b–d). In addition, forest mice have, on average, approximately four additional caudal vertebrae (mean vertebra number: forest, 27.1 (s.d. 0.8) and prairie, 23.2 (s.d. 0.9)), but no difference in vertebra number in other body regions^32^. Together, a linear model including only variation in longest vertebra length and vertebra number accounts for nearly all of the variation in total tail length (*R*^2^ = 0.97, *P* < 0.001). Moreover, vertebral length and number contribute approximately equally to the overall tail-length difference between forest and prairie mice^32^ (Fig. 1d). Together, these data show that heritable differences in total tail length between forest and prairie ecotypes are due to differences in both the length and number of the constituent caudal vertebrae.

### Forest mice outperform prairie mice in arboreal locomotion

**Fig. 2:**
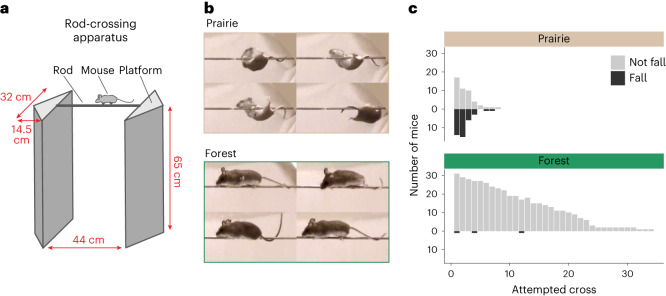
Difference in climbing performance between prairie and forest ecotypes. **a**, A schematic of the rod-crossing apparatus including dimensions (red). **b**, Representative side-view images captured from videos of a rod-crossing assay: prairie (top) and forest (bottom) mice (Supplementary Videos 1 and 2). **c**, The number of mice that fell (dark grey) or did not fall (light grey) on each attempted cross. Prairie mice (top, *n* = 31) fall more often (*P* = 1 × 10^−12^) than forest mice (bottom, *n* = 32).

The repeated association between long tails and forest habitat suggests an adaptive role for the mammalian tail in arboreal lifestyles in mammals, generally (for example, ref. ^36^) and deer mice, specifically refs. ^29–32,34^. Indeed, recent models suggest that a longer tail relative to body size is relevant for balance (that is, controlling body roll) during arboreal locomotion in diverse species^35,37,38^. Thus, theory predicts that long-tailed forest mice will perform better than similar-sized prairie mice in behaviours typical of an arboreal lifestyle. To test this prediction in these subspecies, we used a horizontal rod-crossing assay designed to mimic small-branch locomotion (Fig. 2a). We tested the performance of naive adult mice (forest, *n* = 32 and prairie, *n* = 31) by measuring how often the mice fell from the narrow (0.4 cm diameter) rod and whether they crossed the full length of the rod (44 cm) to another platform (‘completed’ a cross) (Fig. 2b and Supplementary Videos 1 and 2). Forest mice were much less likely to fall: the probability of a forest mouse falling on a given cross is 0.7% (logistic mixed effects model: odds of 0.0073:1), nearly 70 times less than a prairie mouse (48%; odds of 0.906:1; *P* = 7 × 10^−9^) (Fig. 2c). On attempts when a mouse did not fall, forest mice were much more likely to complete a cross (for example, ten times more likely on the first cross; logistic mixed effects model: baseline forest probability of completion 72%, odds of 2.5:1; prairie probability 7.3%, odds of 0.08:1; *P* = 8 × 10^−8^) (Supplementary Fig. 3). Thus, we find that long-tailed forest mice, even after being reared in laboratory conditions and without prior climbing experience, perform better in this rod-crossing assay than short-tailed prairie mice, consistent with a role for tail-length differences in arboreal adaptation in these subspecies.

### Multiple genomic regions contribute to tail-length variation

**Fig. 3:**
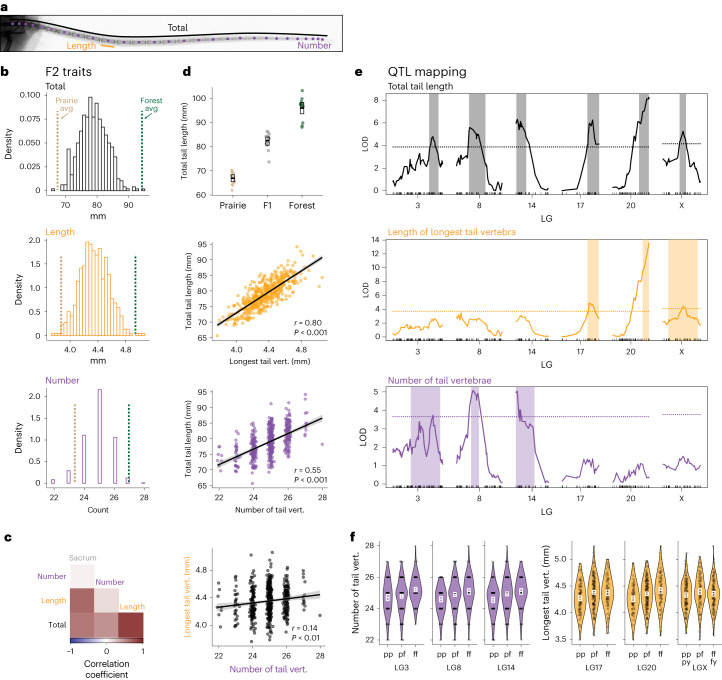
QTL mapping in a forest–prairie F2 intercross for three tail traits. **a**, A tail x-ray highlighting focal measurements: total tail length (black), length of the longest vertebra (orange) and number of vertebrae (purple). **b**, The distributions of tail traits in F2 hybrid mice (*n* = 495). The dashed vertical lines indicate parental trait means (avg): forest (green) and prairie (tan). **c**, Pairwise Pearson correlations between tail traits and sacrum length (a proxy for body size). All correlations are statistically significant at *P* < 0.05 except sacrum versus number. **d**, A plot showing total tail length in each ecotype (*n* = 12 each) and their F1 hybrids (*n* = 14). The boxes show mean and bootstrapped 95% confidence limits of the mean (top). Scatter plots showing the pairwise relationship between the three tail traits in F2 hybrid mice (bottom three plots) (Pearson’s product moment correlation). The lines are linear model best fit; shaded areas indicate 95% CIs. **e**, Statistical association (LOD score) showing significant QTL associations on six LGs for total tail length (top, black), length of the longest caudal vertebra (vert.; middle, orange) and the number of caudal vertebrae (bottom, purple). A shaded rectangle delineates the Bayesian credible interval (0.95 probability coverage) for each significant QTL. The dotted lines indicate genome-wide significance thresholds (*P* = 0.05) as determined by permutation tests. **f**, QTL effects on vertebra number (purple, left three plots) and vertebra length (orange, right three plots) by genotype (pp is homozygous for the prairie allele, pf is heterozygous, ff is homozygous for the forest allele, py is hemizygous prairie male and fy is hemizygous forest male) in F2 mice (*n* = 495) at the peak LOD marker for each QTL. The white boxes show means and bootstrapped 95% confidence limits of the mean.

To characterize the genetic architecture of tail-length variation, we generated a reciprocal genetic cross between forest and prairie mice (*n* = 4 parents; 1 male and 1 female of each ecotype), resulting in 28 F1 hybrids, which then were intercrossed to produce 495 second-generation (F2) hybrids. On the basis of the ecotypic differences and trait correlations in the hybrids, we focused on three tail traits for genetic dissection: total tail length, length of the longest caudal vertebra and number of caudal vertebrae (Fig. 3a,b). The two length traits correlated strongly with body size (Supplementary Fig. 2), so we used sacrum length as a proxy for body size to adjust values in all subsequent analyses of these traits (Methods). In the F2 hybrid mice, vertebra length and vertebra number are both significantly correlated with total tail length (Fig. 3c,d and Supplementary Fig. 2): a linear model with vertebral length and number as the explanatory variables accounts for almost 85% of the variance in total tail length in the F2 hybrid population (*R*^2^ = 0.84). However, vertebral length and number were only weakly correlated with each other (*r* = 0.14, *P* = 2 × 10^−3^; Fig. 3d), suggesting that variation for these two traits is genetically separable. For all three focal traits, F1 hybrid trait values were intermediate between the means of the parental traits (Supplementary Fig. 1) and F2 trait values fell within the mean parental trait values (Fig. 3b). However, for all three tail traits, a few F2 hybrids had trait values similar to the parental phenotypes, consistent with the trait variation being largely oligogenic (Fig. 3b), making these traits amenable to genetic dissection.

We next used interval mapping to localize regions of the genome that influence variation in tail traits in our F2 hybrid population. For total tail length, we identified six significant quantitative trait loci (QTL) that, together in a multiple-QTL model, explain 23.8% of the variance in tail length (Fig. 3e and Supplementary Table 1). The 95% Bayesian confidence intervals (CI) for three of these QTL coincided with those for the three QTL associated with the length of the longest caudal vertebra, which together explained 14.0% of the variance in vertebra length (Supplementary Table 1). The remaining three QTL for total tail length coincided with three QTL that influence the number of caudal vertebrae, these QTL explained 11.7% of the variance in vertebra number (Supplementary Table 1). We also identified two additional weak associations for vertebra length (linkage groups (LGs) 13 and 21), but they did not overlap with QTL for total tail length or vertebra number (Supplementary Fig. 4 and Supplementary Table 1). The distribution of QTL for vertebral length and number on separate chromosomes conforms with the weak correlation between these traits, consistent with vertebral length and number being under independent genetic control.

By examining the effects of each QTL, we estimated the dominance patterns of each allele. We found that alleles inherited from the forest parent exhibit incomplete dominance (Fig. 3f and Supplementary Table 1), with varying degrees of mean dominance-effect estimates ranging from −0.06 to 1.55 (ref. ^39^). In a multiple-QTL model, additive effects of forest alleles at the three vertebra-length QTL ranged from 0.02 mm to 0.10 mm, while the additive effects of forest alleles at vertebra-number QTL were nearly equal (0.26 to 0.29). Thus, an individual with all three forest alleles at the vertebra-length QTL had, on average, a 0.32 mm longer vertebra and at the three vertebra-number QTL, had an average of 1.66 more vertebrae than an animal with prairie alleles at the respective loci. Together, these major-effect QTL explained approximately 33% of the difference of mean vertebra length and 43% of the mean vertebra number difference between forest and prairie ecotypes.

Finally, we performed a sign test that assesses whether the direction of the allelic effects at multiple QTL differ from random expectations^40,41^. We found that for each of the six QTL associated with total tail length, the forest allele effect was always in the expected direction (Fig. 3f), that is, forest alleles result in larger trait values, a pattern that deviates from neutral expectations (*P* = 0.045, Orr’s QTLSTEE; Methods). In addition, a test for directional selection based on the ratio of parental and F2 trait variances also departs significantly from the neutral expectation (*v* = 9.7, *P* < 0.01; *P* < 0.05 for *H*^2^ < 0.73; ref. ^41^). These observations provide additional, independent support for the hypothesis that natural selection favours longer tails in forest deer mice.

### The *Hoxd13* locus is associated with caudal vertebra number

The striking divergence in caudal vertebra number we identified between ecotypes provided an opportunity to explore the genetic and developmental mechanisms that lead to intraspecific segment number evolution. We therefore decided to focus on one tail measure—vertebra number—for further investigation. The number of caudal vertebrae is established in utero (Supplementary Fig. 5). Therefore, to aid in the prioritization of potentially causative genes and to better understand the developmental pathways likely to be important in establishing the vertebra number difference between these ecotypes, we first performed RNA sequencing (RNA-seq) on tail bud tissue spanning the period in which tail somites are forming (‘early’: E12.5, when the first post-hindlimb somites appear, to ‘late’: E15.5, when somitogenesis ends, corresponding approximately to E10.5 and E13.5 in *Mus musculus*^42–44^, respectively) to identify genes that are differentially expressed, even at low levels, between ecotypes (forest, *n* = 18 and prairie, *n* = 17). In a multidimensional scaling analysis, these samples clustered strongly both by ecotype (forest/prairie) and by stage (early/late tail segmentation) (Supplementary Fig. 6a). By comparing expression levels between ecotypes, we found 2,534 and 3,467 protein-coding genes in early and late stages, respectively, that were differentially expressed between forest and prairie embryonic tails (false discovery rate-adjusted *P* < 0.05; Supplementary Fig. 6b). Of these, 1,515 were differentially expressed in the same direction in both stages, while 1,017 were differentially expressed only early on and 1,950 only later (two genes were differentially expressed at both timepoints, but in opposite directions). Thus, perhaps not surprisingly, we found thousands of genes differentially expressed between these ecotypes during a window critical for somitogenesis.

**Fig. 4:**
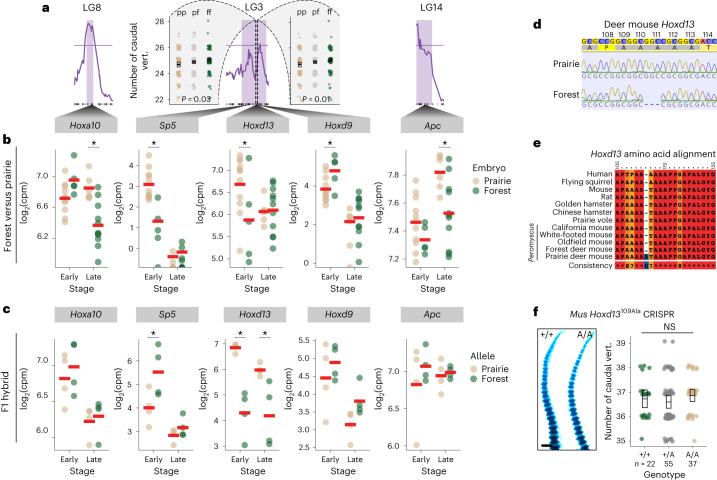
Analysis of candidate genes associated with vertebra number in embryonic tails from forest and prairie mice implicates changes in *Hoxd13 cis*-regulation but not *Hoxd13* amino acid variation. **a**, Three vertebra-number QTL with 95% CIs highlighted (purple) that each contain at least one candidate gene. In the centre, the scatter plots on either side of the LG3 LOD plot show the correlation between vertebral count phenotypes and F2 genotypes at markers flanking the Hoxd cluster (one-way ANOVA; upstream: *F*_2,478_ = 3.69 and downstream: *F*_*2,477*_ = 4.42). The left marker is 679 kb 5′ of *Hoxd13* and the right marker is 624 kb 3′. **b**, RNA-seq-estimated gene expression level (CPM, counts per million) for five top candidate genes in forest (*n* = 18, green) and prairie (*n* = 17, tan) embryos at early (E12.5–13.5) and late (E14.5–15.5) developmental timepoints. **c**, Allele-specific RNA-seq in F1 forest–prairie hybrid embryonic tails (*n* = 8). In **b** and **c**, asterisk indicates empirical Bayes-modulated-*t* Benjamini–Hochberg-adjusted *P* < 0.05 from the linear fit via limma (exact *P* values for **b** are in Supplementary Table 1 and significant *P* values in **c** are 0.03, 4 × 10^−4^ and 0.02 for *Sp5* early, *Hoxd13* early and *Hoxd13* late comparisons, respectively). **d**, Sequence chromatograms showing a portion of *Hoxd13* exon 1 (positions 108–114 a.a.) aligned to the *P.* *maniculatus* *bairdii* reference genome (top). **e**, Profile alignment for a portion of the N-terminal region (*Mus* positions 105–125 a.a.) of HOXD13 in *Peromyscus*, other rodents and human. **f**, Left: examples of transgenic *Mus* wild type and homozygous for the engineered *Hoxd13* CRISPR allele (109Ala) P0 tails stained with alcian/alizarin. Scale bar, 1 mm. Right: caudal vertebral counts for wild type (forest genotype, green), heterozygous (grey) and homozygous (prairie genotype, tan) for the 109Ala allele. NS, one-way ANOVA, *F*_2,111_ = 0.90 and *P* = 0.41; the boxes show mean and bootstrapped 95% confidence limits of the mean.

Variants that are causative for the difference in vertebra number are expected to lie within the three relevant QTL CIs. We therefore next identified the annotated protein-coding genes within each QTL confidence region (*n* = 527, LG 3; *n* = 85, LG8; and *n* = 110, LG14) and intersected these mapping results with the RNA-seq data to identify genes that both fall within QTL CIs and show differential expression. Of the protein-coding genes in these three intervals, we found between 28 and 112 genes in each QTL were differentially expressed during tail development (*n* = 112, LG3; *n* = 28, LG8; and *n* = 28, LG14) (Supplementary Table 2). To identify which of these genes have known effects on tail length, we further prioritized genes that have orthologues with known effects on tail length when manipulated in *Mus* and catalogued in the Mouse Genome Informatics (MGI) phenotype database (Methods and Supplementary Table 3). Of the 155 orthologues included in MGI categories that affect tail length, only five fell within our QTL intervals for vertebra number and also had significant differences in expression levels during embryonic tail elongation: *Sp5*, *Hoxd13*, *Hoxd9* (LG3), *Hoxa10* (LG8) and *Apc* (LG14). Hox genes have known roles in axial patterning, and *Sp5* and *Apc* regulate Wnt signalling; thus, these genes comprise a list of top candidate genes (Fig. 4a).

The causal mutations found within QTL regions that affect expression of candidate genes are expected to act in an allele-specific manner (that is, *cis*-acting). Therefore, we estimated allelic bias in expression using bulk RNA-seq data from F1 hybrid tail bud tissue collected at both early (E12.5) and late (E14.5) tail growth stages (Supplementary Fig. 7). Of the five candidate genes, only *Hoxd13* showed allele-specific expression differences in the same direction observed between the forest and prairie mice (Fig. 4b). While this does not rule out contributions from other genes—there is evidence of *cis* effects acting on *Sp5*, for example, in the opposite direction—it leaves *Hoxd13* as the most likely candidate. Interestingly, the expression difference between the *Hoxd13* alleles in F1s surpassed the difference observed between ecotypes (log_2_ fold change of 0.85 between ecotypes and 2.57 between alleles), suggesting additional *trans-*acting effects that act antagonistically to the *cis*-acting difference. *Hoxd13* has also been shown to be expressed in the tail bud in the laboratory mouse, zebrafish and lizard during axial elongation^45–48^. Together, these data point to *cis*-acting mutation(s) that affect the expression of *Hoxd13* in the developing tail as a strong candidate for contributing to differences in caudal vertebra number.

To confirm that the association between vertebral number and genotype persists in the region near *Hoxd13*, which occurs in a dip in the chromosome 3 QTL logarithm of the odds (LOD) score, we genotyped F2 animals at two markers flanking the gene, one 679 kb 5′ (*n* = 478) and the other 624 kb 3′ (*n* = 477). Genotypes at both markers correlate with vertebral number (one-way analysis of variance (ANOVA); upstream: *F*_2,478_ = 3.69 and downstream: *F*_2,477_ = 4.42) (Fig. 4a). While the maintenance of this association across the Hoxd locus does not rule out contributions of other genes in this interval, it reinforces *Hoxd13* as a strong candidate.

In addition to its expression level, we also compared the entire coding region of *Hoxd13* (1,017 bp) between ecotypes. Although mammalian Hox gene sequences are highly conserved^49^, we found that *Hoxd13* had a 3 bp insertion at amino acid position 109 in the disordered N-terminal region of the protein^50^. The mutation was fixed between our laboratory colonies of forest and prairie mice (Fig. 4c) and resulted in an expansion of a polyalanine tract from four (forest) to five (prairie) residues; expansions of polyalanine tracts in this region of the protein cause hereditary synpolydactyly in humans^51,52^. This 3 bp insertion (or 5-alanine tract) is absent in other *Peromyscus* species, *Mus musculus* and all other rodents we surveyed, and thus appears unique to these prairie mice (*P.* *m.* *bairdii*; Fig. 4d).

We then explored whether this amino acid insertion in *Hoxd13* causes a difference in caudal vertebra number. We first performed a protein variation effect analysis, which predicted that the insertion has a neutral effect on the biological function of the HOXD13 protein (PROVEAN score 0.561) (Fig. 4e). Next, we used clustered regularly interspaced short palindromic repeats–associated protein 9 (CRISPR–Cas9) mutagenesis in C57BL/6 laboratory mice to introduce an extra alanine residue into the native *Mus* 4-alanine tract at position 109 (*Hoxd13*^109Ala^), thereby replicating the prairie allele in *Mus*. Note that the forest allele encodes a protein identical to the native *Mus* HOXD13. When we intercrossed animals heterozygous for the CRISPR edit and counted the number of caudal vertebrae in second-generation pups at birth (P0; *n* = 114), we found no significant effect of the alanine insertion on vertebra number: mice that were homozygous for the 109Ala insertion had a mean of 34.9 vertebrae compared with the wild type 34.7 (one-way ANOVA, *F*_2,111_ = 0.90 and *P* = 0.41, power to detect a difference of 0.52 vertebrae at 0.05 significance of 0.72) (Fig. 4f), noting that our power to detect vertebra number differences in this experiment is influenced by the strength of the phenotypic effect and dominance effects of the insertion. Together, these results suggest that variation in the *Hoxd13* coding region does not affect vertebra number, and instead points to a change in the *cis*-acting regulation of *Hoxd13* expression during a critical time for tail elongation as a likely genetic mechanism.

### Tail development changes correlate with segment number

To determine what developmental mechanisms contribute to differences in caudal vertebra number in deer mice, we compared the developing tail tissues and cell populations of forest and prairie embryos during tail segmentation. Embryonically, vertebrae arise from the sclerotome of the somites, epithelial segments that sequentially bud off at a clock-like rate from the anterior of the PSM^53,54^. Segmentation ends—and the number of vertebrae is determined—when somitogenesis catches up to the tip of the growing tail bud^8,11^. Thus, an increase in somite number can be produced by accelerating the rate of somite production (or slowing the progression of the wavefront) resulting in smaller somites, assuming the same rate of posterior elongation, or alternatively, if the rate of somite formation is constant, increasing the size of the PSM (implying a higher rate of PSM production from the tail bud)^9^.

**Fig. 5:**
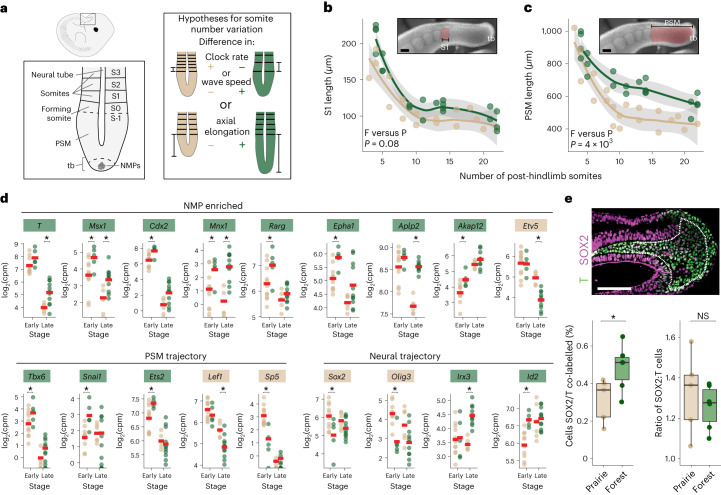
Developmental basis of difference in caudal vertebra number. **a**, Left: a diagram of an E12.5 embryo showing the anatomy of the embryonic tail, including somites (S) and the PSM. Right: hypotheses for how differences in embryonic posterior axis dynamics may produce differences in vertebra number. **b**, The length of the most recently formed somite (S1, pink) across tail segmentation stages (E11.5–E15.5, plotted by number of post-hindlimb somites) measured in fixed specimens of forest (F) (*n* = 20, green) and prairie (P) (*n* = 18, tan) embryos (linear regression, *t* = 1.28, d.f. 2 and *P* = 0.08). tb, tail bud. **c**, The length of PSM (pink) measured in fixed specimens across tail segmentation (E11.5–E15.5) in forest (*n* = 20, green) and prairie (*n* = 18, tan) embryos (linear regression, *t* = 3.05, d.f. 2 and *P* = 4 × 10^−3^). The shaded areas in **b** and **c** indicate 95% CIs of the loess fits. **d**, RNA-seq-estimated transcript counts of genes associated with NMPs as well as PSM and neural fate trajectories that are differentially expressed (* indicates empirical Bayes-modulated-*t* Benjamini–Hochberg-adjusted *P* < 0.05; exact *P* values are in Supplementary Table 2) between forest and prairie embryonic tails at early (E12.5; *n* = 10, forest and *n* = 11, prairie) and late (E14.5; *n* = 11, forest and *n* = 6 prairie) stages of tail development. Gene names are coloured according to the direction of differential expression (green, higher expression in forest and tan, higher expression in prairie). **e**, Top: representative immunofluorescence image from a prairie embryo showing the tail bud mesenchyme (dashed line) in which SOX2 (magenta)- and T (green)-labelled cells were counted. Caudal is to the right. Bottom left: the percentage of co-labelled cells (NMPs) in forest (*n* = 6, green) and prairie (*n* = 5, tan) embryonic tail bud sections at E12.5. Bottom right: the ratio of SOX2-labelled cells to T-labelled cells in sections of tail bud mesenchyme. *P = 0.04 (*t*-test, *t* = −2.4 and d.f. 8.8). The boxes indicate first quartile, median and third quartile, and the whiskers show the range. Scale bars in all micrographs, 100 µm.

To test these hypotheses, we measured the length of both the most recently formed somite (S1) and the PSM in E11.5–E15.5 embryos, following the formation of the first post-hindlimb somites (Fig. 5a). We found that S1 lengths did not differ through time between forest and prairie embryos (linear regression, *t* = 1.28, d.f. 2 and *P* = 0.08; Fig. 5b). Notably, the S1 length differences trended in the opposite direction from expected if somites were produced faster in forest embryos. Moreover, these results are consistent with the rate of somite formation measured in cultured tail bud explants from forest and prairie embryos: we found no significant difference in the rate of somitogenesis (Wilcoxon test, *W* = 31.5 and *P* = 0.45; Supplementary Fig. 8). By contrast, we found that the length of the PSM was significantly different between ecotypes (linear regression, *t* = 3.05, d.f. 2 and *P* = 0.004; Fig. 5c), suggesting different rates of posterior elongation. Specifically, the PSM starts at a similar size but then diverges between ecotypes in the expected direction, that is, larger in forest mice than prairie mice (in embryos with <6 post-hindlimb somites, there is no significant difference in PSM length (Wilcoxon test, *W* = 11 and *P* = 1); for bins 6–12, 12–18 and >18 somites, forest PSM is an average of 129 µm, 189 µm and 111 µm longer, respectively, than prairie PSM; Supplementary Fig. 9). Thus, the comparison of forest and prairie embryos shows that the larger number of caudal vertebrae in adult forest mice is consistent with increased axial elongation rate, resulting in a longer PSM, rather than an increased rate of somitogenesis.

Post-anal PSM size is mediated by regulation of a population of bipotential cells in the tail bud that produce the caudal PSM, the NMPs, a cell population in which *Hoxd13* is expressed during tail elongation in *Mus*^48^. A larger PSM could be produced by either an overall increase in the number of NMP cells or, alternatively, a shift in the balance of NMP fate trajectories towards mesodermal (PSM) to the detriment of neural fates. Indeed, a bias towards the PSM fate in *Mus* results in more segments, whereas a balance tipped towards the neural fate produces fewer^55,56^. To test these alternative hypotheses, we first returned to our transcriptomic data to examine the expression profile of markers enriched in NMP cells as well those for the relevant fate trajectories between forest and prairie mice. Of the genes that were differentially expressed between ecotypes and enriched in NMPs in *Mus* (adjusted *P* < 0.05), eight of nine were more highly expressed in the developing tail buds of forest than prairie mice (Fig. 5d, top) consistent with ecotypic differences in NMP abundance. However, when we examined PSM versus neural fate markers, we did not find evidence for a strong shift towards either the mesodermal or neural fates. In other words, there was no obvious trend in the genes correlated with the NMP fate trajectories (Fig. 5d, bottom): of the five genes highly expressed in the *Mus* PSM trajectory and differentially expressed between ecotypes, three were more highly expressed in forest mice and two in prairie mice, while of the four genes highly expressed in the neural trajectory of *Mus* and differentially expressed between ecotypes, two were higher in forest mice and two in prairie mice. Thus, the RNA-seq data suggest that, while there is no clear shift in gene expression associated with two downstream NMP fates (PSM versus neural), the higher expression of NMP-enriched genes is consistent with a larger pool of axial progenitor cells in forest compared with prairie mice.

To confirm this difference in the number of NMP cells between ecotypes, we counted cells in embryonic tail bud sections immunostained for SOX2 and T, canonical markers for NMP cells, at E12.5 (forest, *n* = 6 and prairie, *n* = 5). We found that a greater proportion of forest tail bud mesenchyme cells are co-labelled with SOX2 and T antibodies than prairie tail buds (*t*-test; *t* = −2.4, d.f. 8.8 and *P* = 0.04; Fig. 5e), consistent with the transcriptomic data, indicating a larger pool of axial progenitors in the forest ecotype. We also compared the ratio of SOX2:T cells in the tail bud mesenchyme of both ecotypes to test for a bias in NMP fates, with the expectation that long-tailed forest mice would have a lower ratio if NMPs were biased towards producing PSM. However, consistent with the transcriptomic data, we did not detect a significant difference in the ratio of SOX2:T immunostained cells (*t* = 0.2, d.f. 7.4 and *P* = 0.9; Fig. 5e), although our power to detect a difference was low. Thus, the results from the transcriptomic and immunohistochemistry experiments together suggest that differences in NMP abundance probably contribute to differences in PSM size between forest and prairie ecotypes.

## Discussion

**Fig. 6:**
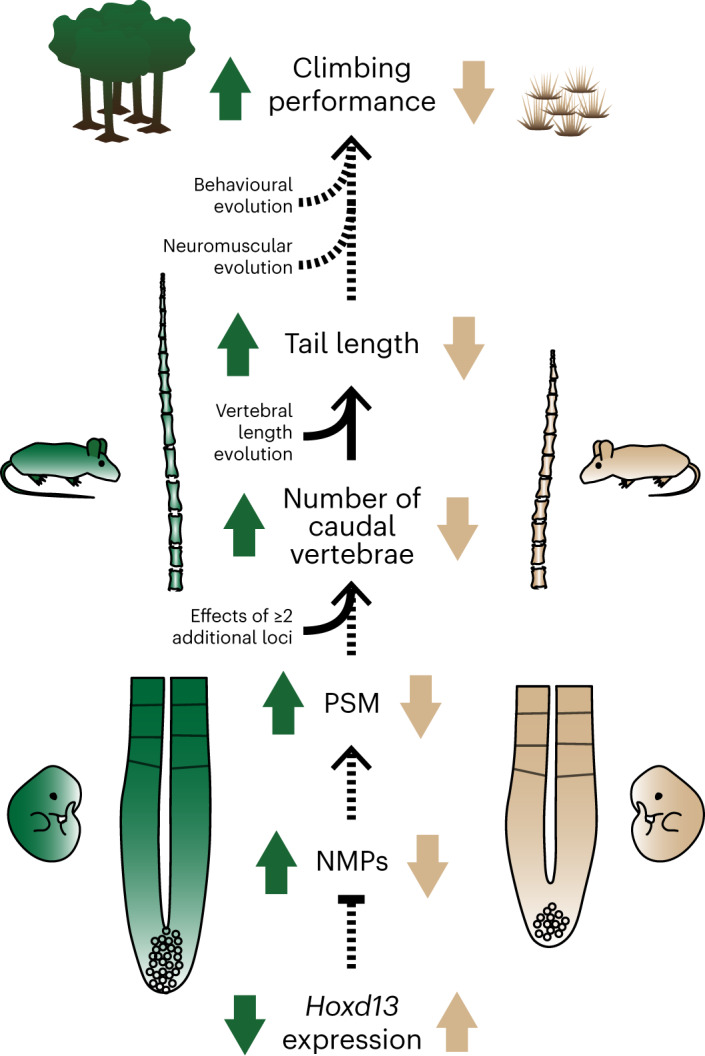
Model highlighting the putative links between the proximate and ultimate mechanisms driving tail-length differences between forest and prairie ecotypes in this study. The black arrows indicate putative (dashed) and causal (solid) relationships between traits at each level of biological organization. The coloured arrows indicate relative directional effects of each trait in forest (green) versus prairie (tan) ecotypes.

Here we investigated both the ultimate and proximate mechanisms driving the divergence in a skeletal trait—tail length—between forest and prairie ecotypes within a single species of deer mice. These tail-length differences are due to changes in both caudal vertebral length and number. In the six genomic regions that are associated with tail-length variation, the forest allele is always associated with longer tails, consistent with natural selection driving trait divergence, probably due to longer tails contributing to at least some aspects of climbing performance in forest environments. In one of these genomic regions lies a strong candidate gene, *Hoxd13*, which shows allele-specific differential expression between forest and prairie embryos during tail elongation. These ecotypes also differ in the size of the tissue from which somites develop as well as its underlying progenitor cell population. Taken together, our results suggest a plausible model for the evolution of vertebra number between deer mouse ecotypes: reduced *Hoxd13* expression maintains the progenitor pool of the tail bud PSM in forest mice, leading to prolonged axial extension, the formation of more somites and ultimately more vertebrae in long-tailed forest compared with short-tailed prairie mice (Fig. 6).

Tail length has long been used as an indicator of habitat occupancy, with longer tails associated with arboreality even among closely related species (for example, squirrels^57^, murine rodents^58^ and field mice^59^). In deer mice, this correlation was thoroughly investigated by Osgood^29^, who described two distinct ecotypes—forest and prairie forms—based on several morphological traits, with differences in tail length being the most conspicuous. Previous studies suggested an important role for tail use in arboreal locomotion by demonstrating that tail amputation in mice dramatically decreases balance^34,60,61^. Based on these data, a clear hypothesis emerged: naturally evolved tail-length differences in deer mice may be important for performance in arboreal climbing^34,62,63^. Recent biomechanical modelling suggests that the longer, heavier tails allow forest deer mice to better control their body roll, as when traversing narrow rods^35^. Indeed, in the subspecies we studied here, we found striking differences in a rod-crossing assay—with forest deer mice falling fewer times and completing more crosses than prairie mice—consistent with experimental studies in other populations and species (for example, refs. ^64–67^). While horizontal climbing on a narrow rod does not capture all the complexities of arboreal locomotion in the wild, deer mice are known to cross narrow twigs in nature^68^; nor does this assay allow us to disentangle the roles of any behavioural (for example, balance and skilled movements) or additional morphological differences (for example, foot size and whisker length) that also may contribute to climbing performance. Nonetheless, these heritable, ecotype-specific differences in rod-crossing ability, in the expected direction, are likely to be at least partly, if not largely, driven by differences in tail morphology.

Genetic mapping allowed us to characterize the genomic architecture underlying total tail length and its constituent components—caudal vertebral length and number—both of which consistently differ between forest and prairie ecotypes across North America^32^. In this species, tail-length differences are largely controlled by six major-effect loci on six different chromosomes. Notably, mapping studies in other wild vertebrates also identified multiple QTL associated with variation in caudal vertebrae (for example, threespine sticklebacks^69,70^ and medaka^71^). As the total variation explained by these six regions together is 24%, this also suggests that many additional loci of small effect were not detectable given the size of our mapping population. Thus, a role for *Hoxd13* would be accompanied by several (possibly many) other genes in establishing vertebra number differences between ecotypes. Of the six loci, three are associated with vertebra length and the other three with vertebra number, consistent with the observation that these traits are not strongly correlated in F2 hybrids. Similarly, artificial selection for increased tail length in replicate lines of laboratory mice resulted in one line with longer vertebrae and the other with more vertebrae^72^. That these traits are genetically separable raises the possibility that the correlation between length and number across deer mice could be due to biomechanical constraints (for example, a trade-off between tail stiffness and flexibility), but modelling does not find support for tail curvature, at least, being strongly influenced by the relative changes in length or number of tail vertebrae in deer mice^35^. Instead, the repeated evolution of coincident length and number differences in deer mice may be due to selection on increased overall tail length by either mechanism when standing variation and/or new mutations are plentiful and exist or appear at roughly the same frequency.

Support for natural selection on tail length in deer mice stems from multiple lines of evidence. First, tail length correlates with habitat, even when corrected for genetic relatedness^32^. Tail-length differences are maintained despite high levels of gene flow connecting forest and prairie populations^73^. Our QTL mapping results provide additional, independent evidence that supports a possible role of selection: all six detected tail-length QTL have allelic effects in the same direction as the overall tail length difference between ecotypes (that is, forest alleles are always associated with longer tails and prairie alleles with shorter tails), a result unlikely to occur by chance. Importantly, these findings are all consistent with the hypothesis of divergent selection acting on tail length: that not only are long tails favoured in forest habitat, but also short tails are favoured in prairie habitat^33^. In the latter case, long tails are probably costly to produce, are a source of heat loss, can be subject to injury and/or may be an additional target for predation^57,62,74^; therefore, without the benefit of, for example, improving climbing performance, the cost of having a long tail outweighs the benefit in terrestrial mice inhabiting open prairie habitats.

Our mapping study also allowed us to narrow in on promising candidate genes contained within the QTL intervals. We found that two of the three QTL influencing vertebra number contain Hox gene clusters: Hoxa and Hoxd. While our approach does not allow us to rule out the involvement of other genes in these intervals, Hox genes are especially intriguing candidates in light of recent studies: in addition to specifying tail identity, the Hox13 paralogues also have been proposed to control axis termination^19,20,56^. First, the most 5′ Hox genes, those of paralogy group 13, are expressed at the tip of the elongating embryonic tail^45,75^, where these genes are known to terminate axial elongation by inhibiting the effects of more anterior Hox genes and repressing Wnt activity^19,76,77^. For example, in *Mus*, loss of *Hoxb13* leads to the formation of supernumerary caudal vertebrae^78^, while its overexpression results in premature truncation of the tail^18^. Moreover, in a study that manipulated Hoxd expression using genomic inversions in *Mus*, an inversion that induced premature *Hoxd13* expression in the tail bud at E10 (deer mouse E12) produced mice with ~2.5 fewer caudal vertebrae than the wild type^79^. Consistent with these studies, we found lower levels of *Hoxd13* in long-tailed forest mice compared with higher levels in short-tailed prairie mice. Thus, together, these genetic data indicate that *Hoxd13* is a strong candidate for contributing to the evolution of caudal vertebra number differences in deer mice: *Hoxd13* is expressed in an appropriate time and place (that is, in the developing tail bud during somitogenesis), it is regulated by *cis*-acting variants as expected for a causative locus identified via QTL mapping and the direction of expression difference between ecotypes is consistent with its known function.

To better understand the developmental mechanisms that ultimately lead to differences in vertebra number, we explored the process of posterior axial development in forest and prairie embryos. Evolution of vertebra number is likely to require changes to the parameters of axial segmentation and/or elongation. Previous studies investigating somite number differences in snakes versus non-snakes^11^, between inbred lines of medaka^71^, and zebrafish *hes6* and *hes7* timing mutants^12,13^ implicated changes to the rate of segmentation: faster somite formation rates produced more, smaller somites. By contrast, here we did not find evidence that forest mice have smaller somites or that somites form at a faster rate, but instead found that forest deer mice develop a larger amount of PSM tissue in the post-hindlimb region. This suggests that forest deer mice may have a faster rate of axis elongation compared with that of prairie mice, but with a similar rate of somite formation. As somitogenesis is thought to end when posterior elongation ceases and the somite formation front ‘catches up’ to the tip of the tail, a longer post-hindlimb PSM is predicted to result in a greater total number of somites^9,80^.

Recent work on axial development has shown how Hox expression can influence, in addition to vertebral identity, the overall length of the vertebral column by regulating posterior axial extension^18,56,81^. This effect is mediated by regulation of progenitor cells, NMPs, that give rise to the posterior PSM; indeed, a single-cell RNA-seq study in *Mus* found that *Hoxd13* is expressed in NMPs^48^. In both mice and fish, posterior Hox genes, especially the Hox13 paralogues, act to regulate this progenitor population, at least in part by inhibiting Wnt and fibroblast growth factor signalling^19,56^ (but see ref. ^47^). Indeed, one prediction of a reduction in *Hoxd13* expression is an increase in Wnt signalling that would sustain the NMP population^19^. Noting that we compare embryos differing at several tail-length loci, we show that *T* and *Cdx2*, which are Wnt targets^82–84^, have higher expression in forest versus prairie mice. As *T* is essential for production of PSM, this provides a potential mechanism by which decreased *Hoxd13* expression in the forest tail bud could result in an elongated embryonic axis. However, not all Wnt target genes (for example, *Lef1* and *Axin2*) show a similar pattern in our data. Thus, the precise details of how the larger NMP population is promoted or maintained have yet to be fully explained. Nonetheless, these results suggest that differences in the size of the axial progenitor pool, probably influenced by *Hoxd13* expression differences, underlie differences in PSM size and thus vertebra number in deer mice.

Developmental geneticists have long known that mutations in Hox genes can affect segmental identity in bilaterian animals^85,86^, although these laboratory-derived homeotic ‘monsters’ are clearly less fit than the wild type. Nonetheless, this potential, along with the correlation of Hox expression patterns with body segments, led many to enthusiastically hypothesize that changes in Hox genes could underlie major morphological shifts in animal body plans in nature^5,7,87,88^. Thus, while HOX protein sequences are conserved due to their pleiotropic roles in development^89–91^, it was unclear whether regulatory changes at Hox loci contribute to segmental evolution in natural populations, especially in vertebrates. Here, we provide evidence that *cis*-acting mutation(s) causing gene expression changes in *Hoxd13* may act through developmental changes to the PSM and its progenitor cells, thus contributing to segment number variation within a single species of deer mouse. Together, this work and parallel work showing *cis*-regulatory changes in *Hoxdb* associated with spine number variation in stickleback fish^92^, demonstrate how Hox genes can contribute to adaptive morphological evolution even on microevolutionary scales in the wild.

## Methods

### Animals

We focused on two subspecies of deer mice, *P.* *maniculatus*, representing the forest (*P.* *m.* *nubiterrae*) and prairie (*P.* *m.* *bairdii*) ecotypes. Forest mice were descendants of 16 wild-caught deer mice that we captured from maple–birch forest in Westmoreland County, Pennsylvania in 2010 (described in ref. ^32^). Prairie mice were descendants of mice obtained from the Peromyscus Genetic Stock Center (University of South Carolina), originally captured in Washtenaw County, Michigan in 1948.

Mice were housed at 23 °C on a 16:8 h light:dark cycle in standard mouse cages (Allentown) with corncob bedding (The Andersons), cotton nestlet (Ancare), Enviro-Dri (Shepherd Specialty Papers) and either a red tube or a red hut (BioServ). Mice were housed in same-sex groups of two to five individuals and provided with water and mouse chow (LabDiet Prolab Isopro RMH 3000 5P75) ad libitum. All breeding colonies and experiments were conducted under and approved by the Harvard Institutional Animal Care and Use Committee protocol 11-05.

### Behavioural assay

To measure an ecologically relevant aspect of climbing performance in which the tail may play a role, we designed a rod-crossing assay, similar to that used by Horner^34^. In brief, we built a custom arena consisting of two 32 cm × 14.5 cm white acrylic platforms (McMaster-Carr), elevated 65 cm above the floor and connected by a 44 cm long, 5/32 inch (0.4 cm) diameter stainless steel rod (Fig. 2a). To start each trial, we placed a naive, adult mouse on the platform for a brief 1 min habituation and then allowed the mouse to voluntarily explore the arena. Trials lasted 5 min after the start of the first cross (defined as when the mouse first placed all four feet on the rod) or for a maximum of 10 min if the mouse never initiated rod crossing. We filmed the trials at 240 fps, 720 × 1,280 pixel resolution, using two GoPro Hero 4 Black cameras mounted on tripods (one top view and one side view). We performed all assays during the light phase, between zeitgeber time 10 and 14 (with zeitgeber time 0 defined as lights on). Between trials, we cleaned the arena with 70% ethanol and allowed it to dry fully. Each mouse was tested once, between 55 and 70 days of age.

For each trial, we manually scored behaviours, including crossing the rod and falling. Specifically, we defined a ‘cross’ as the time between a mouse placing all four feet on the rod and when the last foot was removed from the rod. For each cross, we scored whether the mouse fell (that is, lost all contact with the rod before remounting the platform). In cases in which the mouse did not fall, we noted whether the mouse completed the cross by reaching the other platform (that is, whether the mouse touched the opposite platform at any point during the trial). We report results for all mice that climbed onto the rod at least once during a trial (forest, *n* = 32 of 35 complete trials and prairie, *n* = 31 of 46 complete trials).

If a mouse fell or jumped from either the rod or the platform, the experimenter stopped the assay and replaced the mouse on the platform. If a mouse jumped from the platform more than five times during a trial, the trial was discontinued and not analysed further (forest, *n* = 5 and prairie, *n* = 5).

#### Statistical analysis of behaviour

We analysed behaviour data using generalized linear mixed models (family ‘binomial’, lme4 package v. 1.1 in R v. 3.6.2; refs. ^93,94^), including data for only the first eight cross attempts, as no prairie mice crossed more than eight times during the trial while forest mice crossed up to 34 times. Each response variable was binary (‘fell’ or ‘completed’). We fit models with the following sets of fixed effects: cross index alone (null model, cross number is included to account for possible effects of experience), ecotype alone (that is, no effect of experience), additive effects of ecotype and cross, or an interaction between ecotype and cross (that is, different effects of experience in the two ecotypes). Each model also included individual as a random effect. We compared these models using likelihood ratio tests (implemented in the ANOVA function, stats package).

### Genetic cross

#### Forest–prairie F2 hybrid intercross

To produce a genetic mapping population, we established a reciprocal intercross between two ecotypes: forest (*P.* *m.* *nubiterrae*) and prairie (*P.* *m.* *bairdii*). The mapping cross consisted of two families, each founded by two animals: family ‘0’: female *bairdii* × male *nubiterrae* and family ‘1’: female *nubiterrae* × male *bairdii*. Cross parents were siblings. We established 14 F1 breeding pairs, which when intercrossed produced 495 F2 hybrids (family 0, *n* = 211 and family 1, *n* = 284) for analysis. F2 hybrids were killed between ages 70 and 300 days and were measured for gross morphology (total length, tail length and body mass).

### Skeletal measurements

We measured lengths of limb bones and tail bones in 12 forest, 12 prairie, 14 F1 hybrid animals and 495 F2 hybrid animals from x-ray radiographs. We used a digital x-ray system (Varian Medical Systems, Inc.) in the Harvard Museum of Comparative Zoology Digital Imaging Facility to obtain radiographs of whole specimens mounted such that the plane containing the anterio-posterior and medio-lateral axes was parallel to the imaging plane. We measured all traits with Fiji/ImageJ^95^ and we included a standard to determine scale. In total, we measured up to 32 sacral and caudal vertebrae, maximum caudal vertebra length and caudal vertebra number, as well as total sacrum length and total tail length (Supplementary Fig. 1).

Most bone-length traits were correlated with body size in our cross; therefore, we corrected for body size using linear regression on sacrum length. Sacrum length, a section of the vertebral column that is anterior to the caudal vertebrae and does not significantly differ in length between ecotypes (Wilcoxon test, *W* = 66 and *P* = 0.75), represents a standard for body size (sacrum length versus body mass: in F2s, Pearson’s *r* = 0.54, 95% CI 0.48–0.60 versus ruler-measured body length: *r* = 0.64, 95% CI 0.58–0.69). We corrected for body size by regressing raw trait measures against the sum length of the four sacral vertebrae, and added the residuals from that regression to the trait mean to align the corrected measurements in the ranges of the raw measurements.

To describe variation in tail length, we used three summary statistics: (1) the number of caudal vertebrae (all vertebrae posterior to the four sacral vertebrae), (2) the length of the longest vertebra in the tail and (3) the total length of the tail, measured from the x-ray radiographs (Fig. 3a). We explored the pairwise correlations among traits in the F2 animals and conducted a principal component analysis (as implemented in the ‘principal’ function in the psych package in R; refs. ^94,96^) using measurements with the standard deviations for each trait scaled to 1 and centred the means of each trait to 0. The first three components account for 68% of the variance in sacral and caudal vertebral lengths.

### Genotyping and linkage map construction

We genotyped parent, F1 and F2 animals using double-digest restriction site-associated DNA sequencing^97^. Briefly, we extracted genomic DNA from alcohol-preserved liver tissue with the AutoGenprep 965 (AutoGen), digested it with EcoRI and MspI (New England Biolabs) and ligated end-specific adapters, P1 and P2 that include individual barcodes and biotin labels, respectively. Next, we combined samples into 48 individual pools and size-selected each pool to 216–276 bp using a Pippin Prep (Sage Science), after which we used streptavidin beads (Dynabeads M-270, Life Technologies) to eliminate fragments without P2 adapters. We PCR-amplified these pools (ten cycles) with an indexed primer. Using a TapeStation (Agilent), we quantified the mass of these pools (range from 0.7 to 5.0 nM) and combined them in equimolar ratios. Finally, we sequenced these pools in 150 bp paired-end rapid runs on an Illumina HiSeq 2500 to ~600 K reads per sample.

We processed the sequence reads using custom Python software^98^. In brief, this software used Stampy to map merged paired-end reads to the *P.* *maniculatus* genome scaffolds (GCA_000500345.1) and then combined reads by individual into BAM files with Picard^99^. We then used the Genome Analysis Toolkit^100,101^ to call variants with UnifiedGenotyper. From 4.3 × 10^8^ raw reads, this analysis produced 1.1 × 10^7^ called variants. We hard filtered these variants for those that were fixed between the prairie and forest parents of the cross, those with QD > 5, GQ > 30 and those present in more than half the F2 individuals (using HTSeq^102^). This filtering produced 4,296 variants, which we used to construct a linkage map using R/qtl, closely following the procedure outlined by Broman and Sen^103^. The resulting map had 24 LGs, corresponding to the haploid number of chromosomes in *P.* *maniculatus*^104^, comprising 2,618 markers with an average spacing between markers of 0.7 cM and a maximum spacing of 23.1 cM.

We performed additional genotyping at two markers flanking the Hoxd cluster in 489 F2 animals. We used custom Taqman single nucleotide polymorphism genotyping assays (ThermoFisher; oligonucleotide sequences in Supplementary Table 4) to genotype fixed variants at chr4:51286795 and chr4:52593788 (Pman2.1.3; GCA_003704035.3) on an Eppendorf Mastercycler Ep Gradient S Realplex 2. The PCR conditions were as follows: 10 min at 95 °C; 40 cycles of 10 s at 95 °C and 60 s at 60 °C (measurement taken at the 60 °C step).

### QTL mapping

We used R/qtl^103^ to identify regions of the genome in which genetic variation was statistically associated with variation in skeletal traits. For all bone-length traits, we performed standard interval mapping with the extended Haley–Knott method (‘ehk’ in the R/qtl scanone function) including sex, age and sacrum length as additive covariates. As the number of caudal vertebrae (count) was not continuous and not normally distributed (Shapiro–Wilk test: *W* = 0.90 and *P* < 1 × 10^−15^), we used the non-parametric method for mapping. We used permutation tests (*n* = 1,000 permutations for autosomes and *n* = 26,312 for the X chromosome) to determine significance thresholds for each trait^105^.

To assess the effect sizes of each QTL and the amount of variance each locus explained, we used multiple-QTL models and drop-one analysis in R/qtl. Using the *P* < 0.05 significance thresholds as determined by permutation tests, we fit models for each trait with the genotypes at markers with the highest LOD scores in each significant QTL as explanatory variables as implemented in fitqtl. The models for length traits include sex, age and sacrum length as additive covariates.

We assessed evidence for selection on tail length using the direction of QTL effects with QTLSTEE^40^ and with the ratio of parental and F2 variances with the *v*-test^41^. For the *v*-test, we used a conservative assumption of additivity (*c* = 2) and estimated *H*^2^ using parental, F1 and F2 variances^106^.

### Embryo collection

We generated embryos of approximate ages (E11.5–E15.5) from each ecotype. As *Peromyscus* mice experience postpartum oestrus^107^, we set the date of conception as the birth date of a female’s last litter and then confirmed these ages using a developmental time series of *Peromyscus*^43,44^.

### RNA-seq of embryonic tail tissue

We dissected post-anal tail tissue from 35 embryos (forest, *n* = 18 and prairie, *n* = 17) at Theiler stages 15–20 (E12.5–E15.5), timepoints relevant to tail somitogenesis^42^. We extracted total RNA using the PicoPure RNA Isolation kit (ThermoFisher Scientific) and constructed RNA-seq libraries using PrepX poly-A and library prep kits on an Apollo 324 System, following the manufacturer’s protocol. We sequenced libraries on two lanes of 150 bp paired-end runs on an Illumina HiSeq 2500 to ~30 million reads per sample.

To measure allelic expression bias in F1 hybrid embryos, we dissected embryonic tails at E12.5 (*n* = 4) and E14.5 (*n* = 4) and extracted RNA using 50 µl Direct-zol (Zymo Research) following the manufacturer’s protocol and used the same library preparation procedures as for the parental samples. We sequenced libraries on one 150 bp paired-end run on an Illumina NovaSeq SP flowcell to ~45 million reads/sample.

We assessed differential expression using an established workflow, following ref. ^108^. Briefly, we trimmed reads using Cutadapt^109^ via Trim Galore!^110^ and mapped reads to the *P.* *maniculatus* genome (Pman2.1.3; GCA_003704035.3) (forest and prairie libraries) or a custom hybrid genome created from variants called from RNA-seq reads (F1 libraries) using STAR aligner^111^. Eighty-five per cent of annotated transcripts in the hybrid genome have at least one variant that allowed allele assignment, including our top five candidate genes. We quantified transcripts using RNA-seq by Expectation-Maximization^112^ and used edgeR^113^and limma-voom^114^ to compare transcript abundance between ecotypes or between alleles, at both early (E12.5–13.5) and late (E14.5–15.5) stages. When comparing allelic bias in F1 embryos, we used the RNA-Seq by Expectation-Maximization gene-level count estimates, effectively summing over all transcripts for a gene and ignoring any isoform-specific effects. For all libraries, we normalized using the trimmed mean of *M*-values method, as implemented in edgeR, and ranked differentially expressed genes by the empirical Bayes method in limma.

### Identification of candidate genes

To prioritize candidate genes related to skeletal variation within QTL intervals, we first calculated 95% CIs for each QTL using the bayesint function in R/qtl. We extracted names of genes in the QTL intervals from the *P.* *maniculatus* genome (GCA_000500345.1) annotation and used the resulting list of gene names to cross-reference with alleles from the MGI Mammalian Phenotype Browser^115^ that have ‘limb/digits/tail’ phenotypes (Supplementary Table 3).

### CRISPR–HDR for HOXD13 amino acid mutation

To test the effect of *Hoxd13* amino acid mutations on tail development, we conducted a CRISPR–Cas9 homology-directed repair (HDR) experiment in *Mus*. Specifically, we designed a guide RNA and HDR template to insert a single alanine into the *Mus Hoxd13* locus at amino acid position 109 (*Hoxd13*^A109^). The sequences of the synthesized guide RNA (Synthego) and single-stranded HDR template (Integrated DNA Technologies) are provided in Supplementary Table 4. These were injected along with Cas9 protein (Integrated DNA Technologies) into C57BL/6J zygotes by the Harvard Genome Modification Facility.

We amplified and sequenced the edited allele (primer sequences in Supplementary Table 4) from tail-tip DNA and assessed editing efficiency using the Synthego ICE tool (ice.synthego.com). We mated the three males and three females with the highest editing efficiency to wild-type animals and then intercrossed siblings to produce F2 offspring (+/+, *n* = 22; +/d13^A109^, *n* = 55; d13^A109^/d13^A109^, *n* = 37). A successful edit destroys a PstI restriction site, so we genotyped P0 F2s using the same primers followed by PstI restriction digestion of the resulting amplicon. To confirm that the correct edit was made, we sequenced *Hoxd13* exon 1 in a subset of F2 animals (*n* = 4 homozygotes for each allele from each family, 24 total); we did not find any off-target mutations in these sequences.

#### Postnatal vertebral counts

We used whole-mount bone/cartilage staining to compare caudal vertebra counts in laboratory-reared neonatal (P0) pups of forest (*n* = 6) and prairie (*n* = 6) ecotypes, and of +/+ and *Hoxd13*^A109^*/Hoxd13*^A109^ CRISPR–HDR (*n* = 114) *Mus* F2s. We stained bone and cartilage with alizarin/alcian following Rigueur and Lyons (2014) and counted all recognizable segments in the tail, including non-ossified cartilage condensations at the caudal tip (Fig. 4e and Supplementary Fig. 5a). Investigators were blind to ecotype/genotype when counting segments.

### Measurement of PSM and somite lengths

To compare tissue dimensions in fixed embryos, we killed females and dissected embryos in phosphate-buffered saline (PBS), then fixed the embryos in phosphate-buffered 4% formaldehyde for 14–24 h at 4 °C. We stained whole embryos with 1 µg ml^−1^ 4′,6-diamidino-2-phenylindole (DAPI) for 30 min and photographed them with a Zeiss mRc camera on a Zeiss steREO Discovery V.12 dissecting microscope that was scale calibrated. We used the linear measurement tool in Fiji/ImageJ^95^ to measure somite and PSM lengths. We analysed these data in R (ref. ^94^) and made plots using ggplot2 (ref. ^116^).

### Embryonic tail explant culture and time-lapse imaging

To obtain precise measurements of segmentation and axial extension parameters, we cultured posterior embryonic tissues and time-lapse imaged them. We dissected E12.5–E15.5 embryos in Dulbecco’s modified Eagle medium that was pre-warmed to 37 °C, dissected the portion of the embryo caudal to the hindlimb bud and transferred that explant to an uncoated Mat-Tek glass-bottomed culture dish also containing pre-warmed Dulbecco’s modified Eagle medium. We then transferred the dish containing explant to a culture chamber at 37 °C with a humidified carbon dioxide (5%) line on a Zeiss Cell Observer (Harvard Center for Biological Imaging). We used Zen 2012 (Zeiss) software to take images every 10 min over a 12–14 h period while the explant formed somites and underwent axial extension. We took a *Z*-stack for each timepoint and used the ‘Extended Depth of Focus’ function in Zen to collapse the stack into a single image for each timepoint. From these time-lapse movies, we obtained basic information about the timing of segment formation using Fiji/ImageJ^95^ to mark the formation of somite boundaries on individual frames. All explants settled slightly during the first 90–120 min; for all time-lapse movies, we discarded the first 12 frames.

### Immunostaining and cell counting

We dissected embryos from pregnant female forest and prairie mice (forest, *n* = 6 and prairie, *n* = 5), and fixed embryos in phosphate-buffered 4% formaldehyde for 14–24 h at 4 °C. We rinsed them with PBS, then embryos were graded through 10% sucrose/PBS (1 h at 20 °C), 30% sucrose/PBS (overnight at 4 °C) and then mounted in optimal cutting temperature medium and frozen. We cryosectioned tails in the sagittal plane at 14 µm per section, then immunostained with anti-Sox2 (R&D Systems MAB2018; 1:500), anti-Brachyury/T (R&D Systems AF2085; 1:500) and fluorophore-conjugated secondary antibodies (anti-mouse-AlexaFluor555 and anti-goat-AlexaFluor488; 1:500; ThermoFisher), each overnight at 4 °C. We counterstained with 1 µg ml^−1^ DAPI (30 min at 20 °C) and imaged sections with a Zeiss LSM710 confocal microscope with a Plan Apo 20×/0.8 Air differential interference contrast (DIC) II objective. We outlined regions of tail bud mesenchyme (for example, Fig. 5e) in a single section per embryo closest to the midline and counted by hand the total number of DAPI-labelled nuclei, SOX2-positive cells, T-positive cells and SOX2/T co-labelled cells in this region. Investigators were blind to ecotype when counting cells.

### Reporting summary

Further information on research design is available in the Nature Portfolio Reporting Summary linked to this article.

### Supplementary information

Supplementary Information Supplementary Figs. 1–9.

Reporting Summary

Supplementary Tables 1–4 Supplementary Table 1. Tail-length QTL. Supplementary Table 2. Annotated genes in QTL intervals with differential expression in embryonic tails. Supplementary Table 3. Phenotypes from MGI database. Supplementary Table 4. Nucleotide sequence of PCR primers, CRISPR–HDR template, CRISPR guide and Taqman genotyping assays.

Supplementary Video 1 Representative first cross attempt by a forest mouse (*P. maniculatus nubiterrae*).

Supplementary Video 2 Representative first cross attempt by a prairie mouse (*P. maniculatus bairdii*).

## Data Availability

The raw and processed forest, prairie and F1 RNA-seq data have been uploaded to NCBI GEO, accessions GSE191280 and GSE191330, respectively. Measurement data (forest, prairie, F1, F2 and embryo, *Hoxd13* CRISPR–HDR *Mus*), rod-crossing data and F2 cross genotypes are available on Data Dryad (10.5061/dryad.jsxksn0gr).
