# Supplementary material for: Adaptive tail-length evolution in deer mice is associated with differential Hoxd13 expression in early development
Source: Nat Ecol Evol. 2024 Feb 20;8(4):791–805. doi: 10.1038/s41559-024-02346-3 (PMC11009118; doi:10.1038/s41559-024-02346-3)
Supplement: Supplementary file 1 — Supplementary Figs. 1–9. [file 41559_2024_2346_MOESM1_ESM.pdf]

nature ecology & evolution

Article

https://doi.org/10.1038/s41559-024-02346-3

Adaptive tail-length evolution in deer mice is associated with differential *Hoxd13* expression in early development

In the format provided by the authors and unedited

SUPPLEMENTARY FIGURE LEGENDS

Supplementary Figure 1. Skeletal trait measurements in parental and hybrid mice. (Related to Figs. 1 & 3) **A.** Total tail length, length of longest vertebra, and number of tail vertebrae in lab-reared prairie (n = 12, tan), F1 (n = 14, gray), and forest (n = 12, green) mice. Boxes show mean and bootstrapped 95% confidence limits of the mean. **B.** Scatter plot showing the relationship between sacral vertebrae length and body length in prairie (n = 12, tan) and forest (n = 12, green) with linear model fit lines. **C.** Example of x-ray image used to measure vertebra lengths for sacral (s) and caudal (ca) vertebrae. **D.** Distributions of vertebrae length (for each vertebra, s1–4, ca1–28) in F2 hybrid mice (n = 495). Parental mean values are indicated by lines: forest (green) and prairie (tan).

Supplementary Figure 2. Correlations among tail traits in F2 hybrid mice. (Related to Figs. 1 & 3) **A.** Heatmap of pairwise Pearson correlation coefficients between pairwise tail traits in all F2s (n = 495). All traits except vertebra number are corrected for sacral vertebrae length (see Methods). **B.** Scatter plots showing the pairwise relationships between sacral vertebrae length, body mass, and body length in F2 hybrid mice with linear model best-fit lines. Shaded areas indicate 95% confidence intervals. **C.** Heatmap of vertebra length loadings from PCA showing that vertebra length traits in different tail regions load onto the first three PCs. Percent variance accounted for by each PC indicated above each column. Abbreviations: s, sacral vertebra; ca, caudal vertebra. * = $p < 0.05$, ** = $p < 0.01$, *** = $p < 0.001$.

Supplementary Figure 3. Probability of mice completing a rod crossing. (Related to Fig. 2) The baseline odds (i.e., on the first cross attempt) of a prairie mouse (tan) crossing fully are 0.08:1 (probability 7.3%) and for a forest mouse (green), 2.51:1 (probability: 72%) (z = 5.36, p = 8e-8 for effect of ecotype). For each additional cross, the log-odds of a full cross increase by 0.27 (z = 3.09, p = 2e-3) for both ecotypes. (Logistic mixed effects model; formula: *complete_cross ~ ecotype + cross*).

Supplementary Figure 4. Genome-wide QTL maps for three focal tail traits. (Related to Fig. 3) Statistical association (LOD, or log of the odds, score) between genotype and phenotype across all linkage groups for **A.** total tail length (black), **B.** length of longest caudal vertebra (orange), and **C.** caudal vertebrae number (purple). Horizontal lines indicate genome-wide significance thresholds (p = 0.05) as determined by permutation tests (see Methods). For details of each QTL, see Supplementary Table 1.

Supplementary Figure 5. Difference in vertebra number between forest and prairie mice is present at birth. A. Examples of postnatal day 0 (P0) tail skeletons stained with alizarin/alcian with caudal vertebra number given. **B.** Caudal vertebra number for P0 forest (green) and prairie (tan) mice (n = 6 for each ecotype). Red lines indicate means. Vertebra number differs significantly (two-sided Wilcoxon test; W = 0, p = 4e-3). Scale bars = 500µm.

Supplementary Figure 6. RNA-seq-estimated differential expression between forest and prairie embryonic tail tissues. (Related to Figs. 4 & 5) **A.** Multidimensional scaling (MDS) plot of bulk RNA-seq libraries (forest, green, n = 18; prairie, tan, n = 17) based on top 500 most highly expressed genes in embryonic tails from early (E12.5–13.5, circle) and late (E14.5–15.5, triangle) embryonic stages of tail elongation. Top panel: Examples of early- and late-stage embryonic tails, tailbud on the right. Scale bar = 100 µm. **B.** Volcano plots showing differentially expressed genes between forest and prairie samples at early and late stages of tail elongation. Blue dots indicate empirical Bayes-modulated-t Benjamini-Hochberg-adjusted $p <$ 0.05 from the linear fit *via* limma. Gene name labels indicate differentially expressed genes associated with NMPs (black text) or candidates from QTL intervals (red text). logFC = $\log_2$ fold change, forest - prairie.

Supplementary Figure 7. RNA-seq-estimated allele-specific differential expression in forest-prairie F1 hybrids. (Related to Fig. 4) **A.** Multidimensional scaling (MDS) plot of bulk RNA-seq F1 forest-prairie hybrid libraries based on the top 500 most highly expressed genes in embryonic tails showing forest (green) and prairie (tan) alleles from early (E12.5, circle) and late (E14.5, triangle) embryonic stages of tail elongation. **B.** Bar plot showing the number of fixed differences between prairie and forest alleles per gene, which were used to assign reads to each ecotype. **C.** Volcano plots showing genes with allele-specific expression at E12.5 and E14.5. Blue dots indicate genes with significant allele-specific expression (empirical Bayes-modulated-t Benjamini-Hochberg-adjusted $p < 0.05$ from the linear fit *via* limma). Gene name labels show genes that both are in QTL intervals and have MGI tail phenotypes. logFC = $\log_2$ fold change, forest allele - prairie allele.

Supplementary Figure 8. Timing of somite formation in embryonic tail explants. (Related to Fig. 5) **A.** Still images from time-lapse imaging of an E13.5 prairie embryonic tail explant. Recently formed somite borders are indicated by white arrows. Time stamps shown. **B.** Mean time between appearance of somite borders from tail explant cultures (forest, n = 9; prairie, n = 9; E12.5–15.5) shows no detectable difference in rate of somite formation (two-sided Wilcoxon test; W = 49.5, p = 0.45). Scale bar = 100µm.

Supplementary Figure 9. Binned somite- and PSM-length measurements. (Related to Fig. 5) **A.** Length of the most recently formed somite (S1) across embryonic tail development (E11.5–E15.5; as determined by number of post-hindlimb somites) measured in fixed specimens of forest (n = 20, green) and prairie (n = 18, tan) embryos. Data are the same as in Fig. 5B but in 6-somite bins. **B.** Length of presomitic mesoderm (PSM) measured in fixed specimens of forest (n = 20, green) and prairie (n = 18, tan) embryos. Data are the same as in Fig. 5C but in 6-somite bins. * = $p < 0.05$ (two-sided Wilcoxon test); Boxes indicate first quartile, median, and third quartile; whiskers show range.

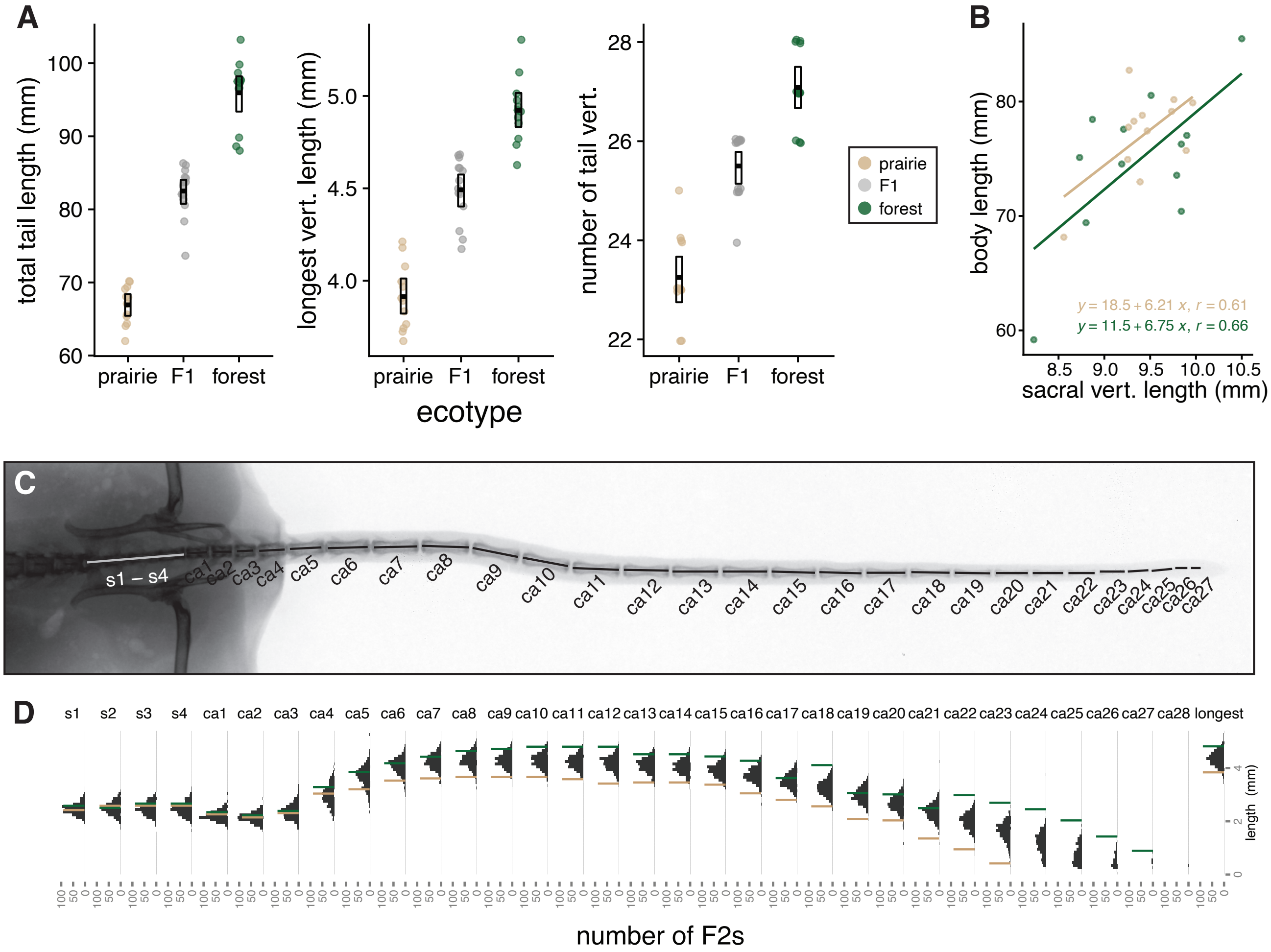

Supplementary Figure 1

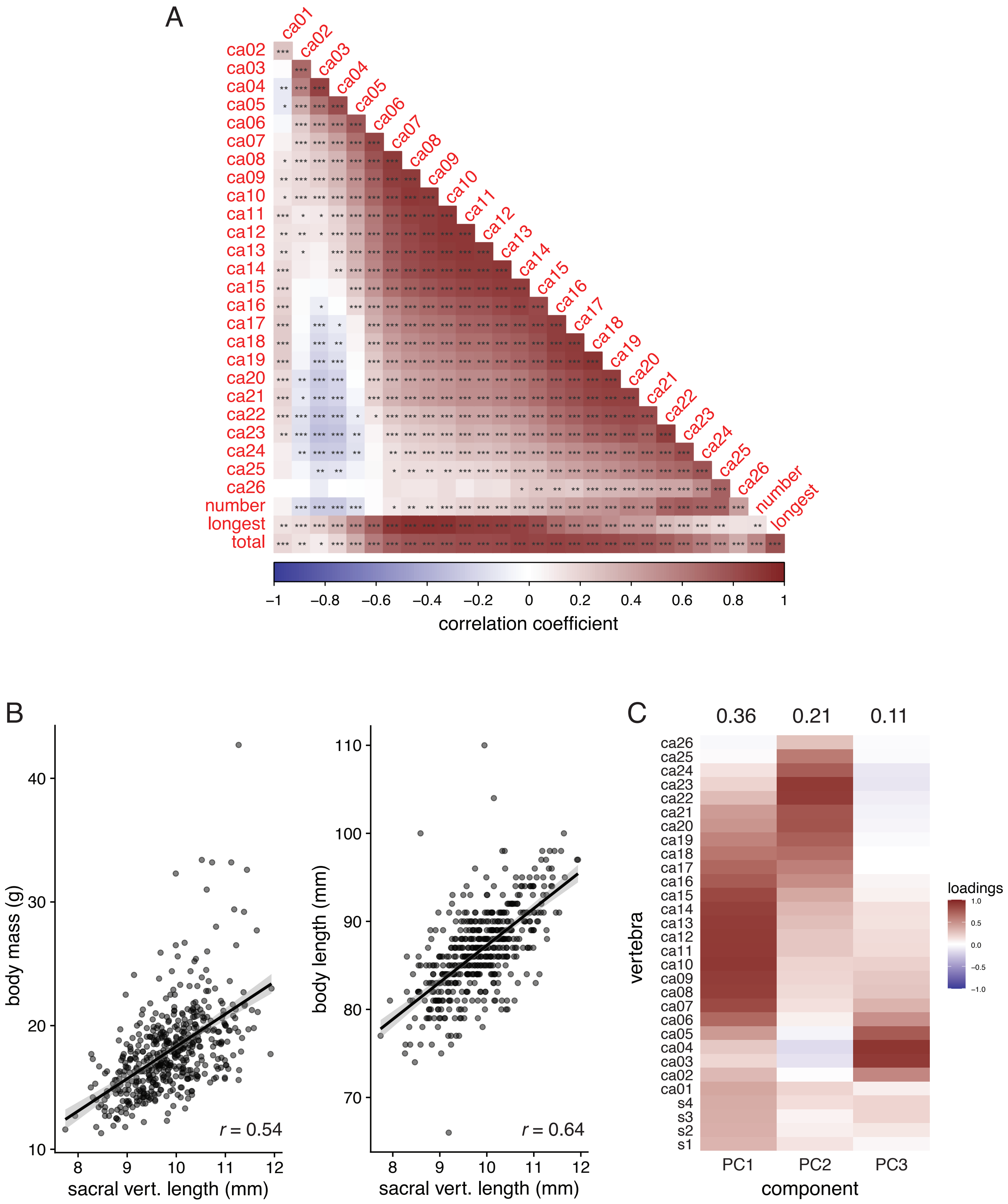

Supplementary Figure 2

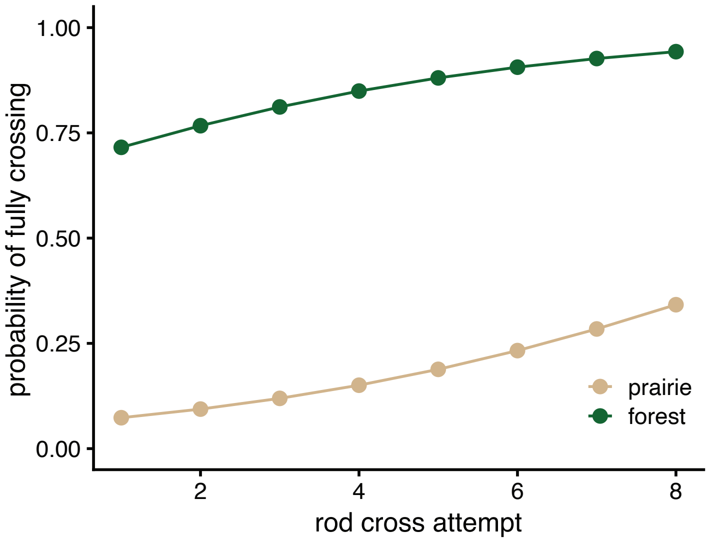

Supplementary Figure 3

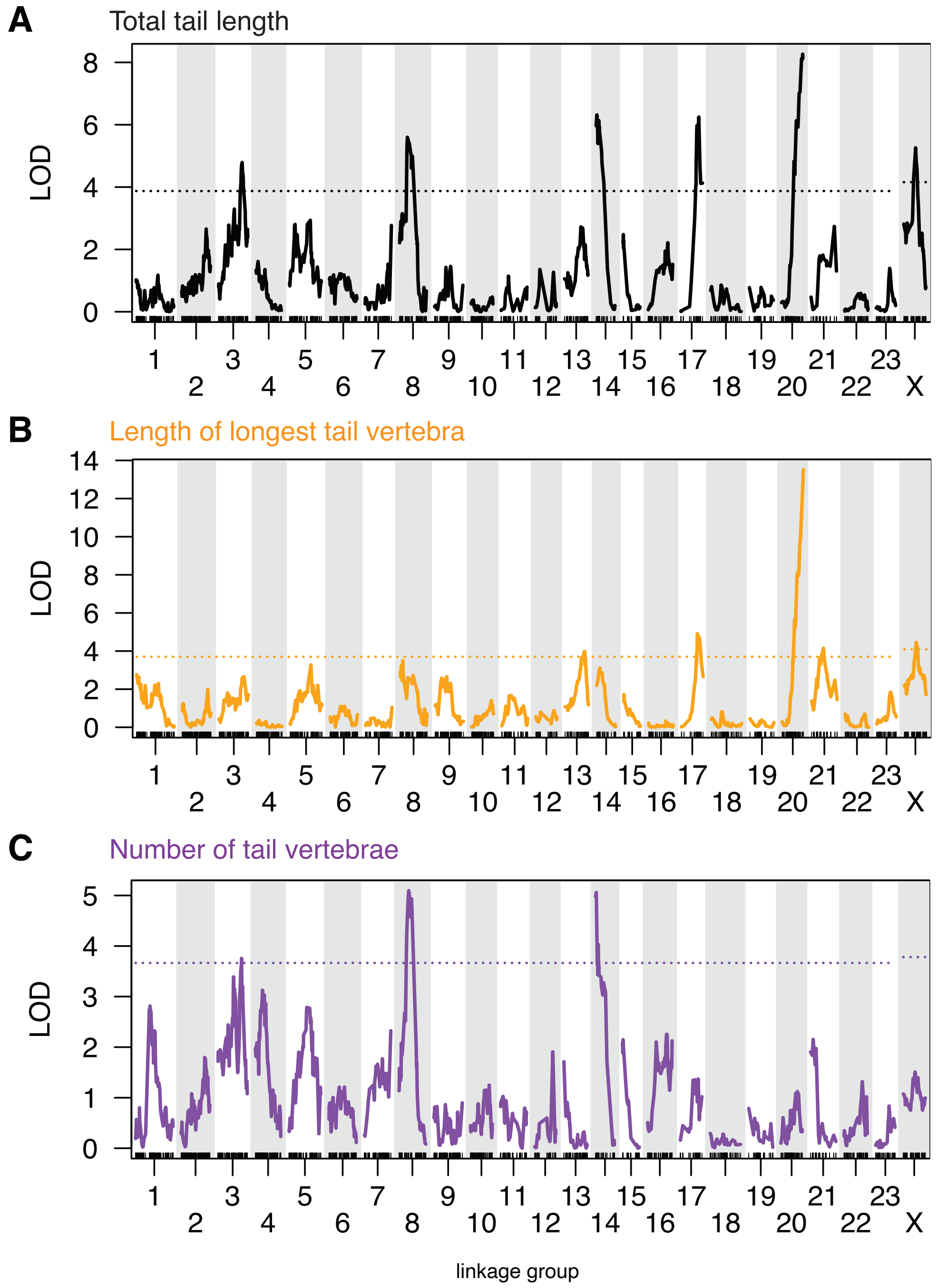

Supplementary Figure 4

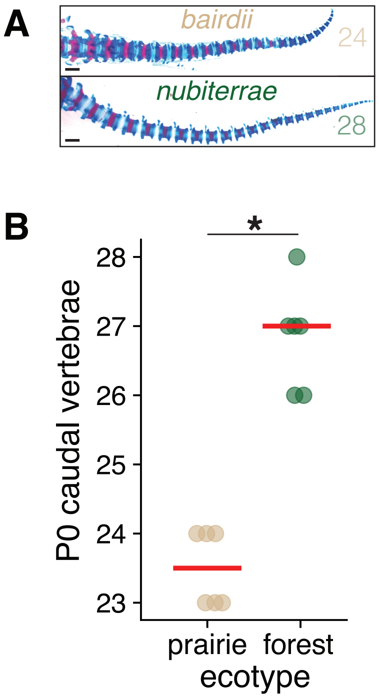

Supplementary Figure 5

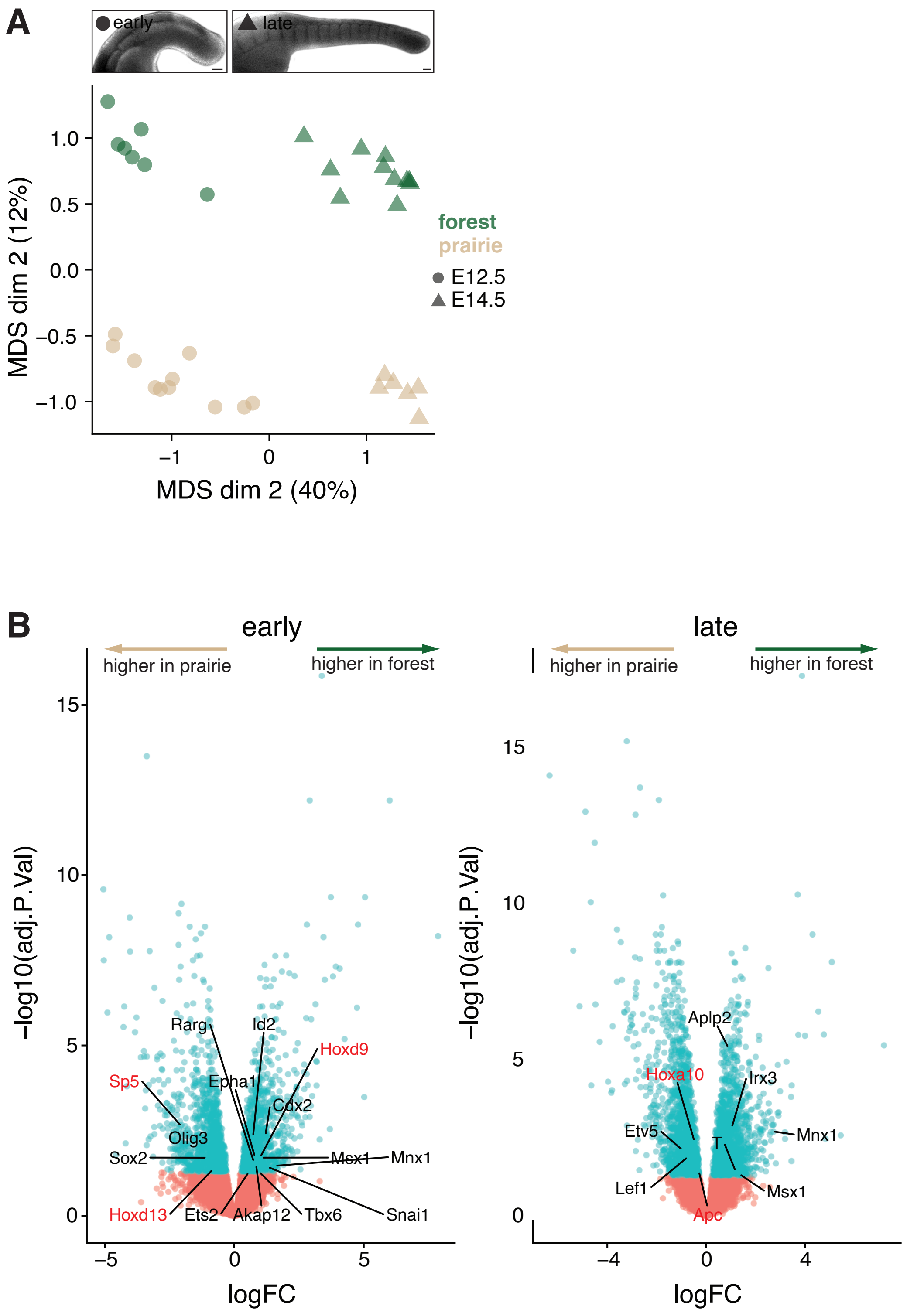

Supplementary Figure 6

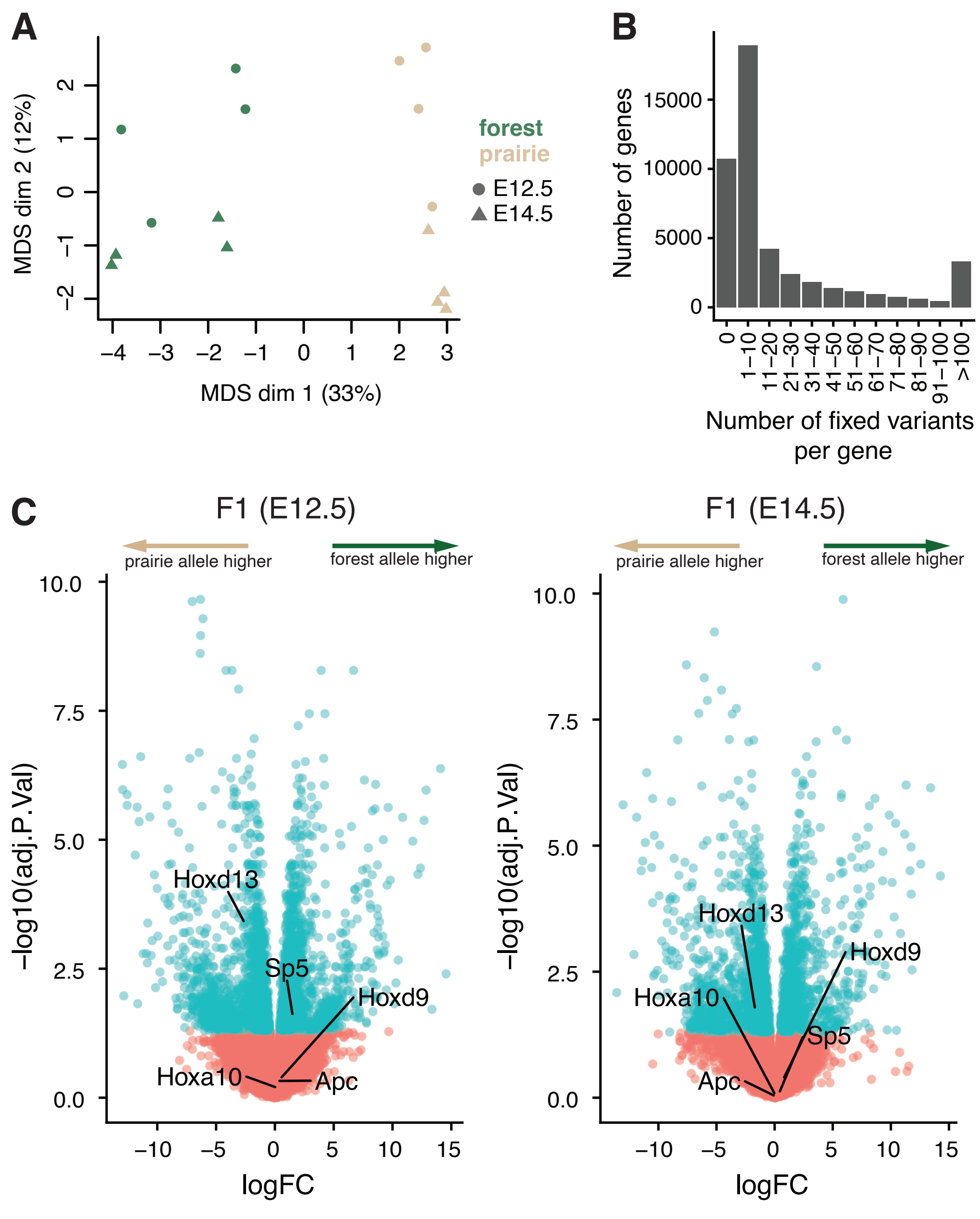

Supplementary Figure 7

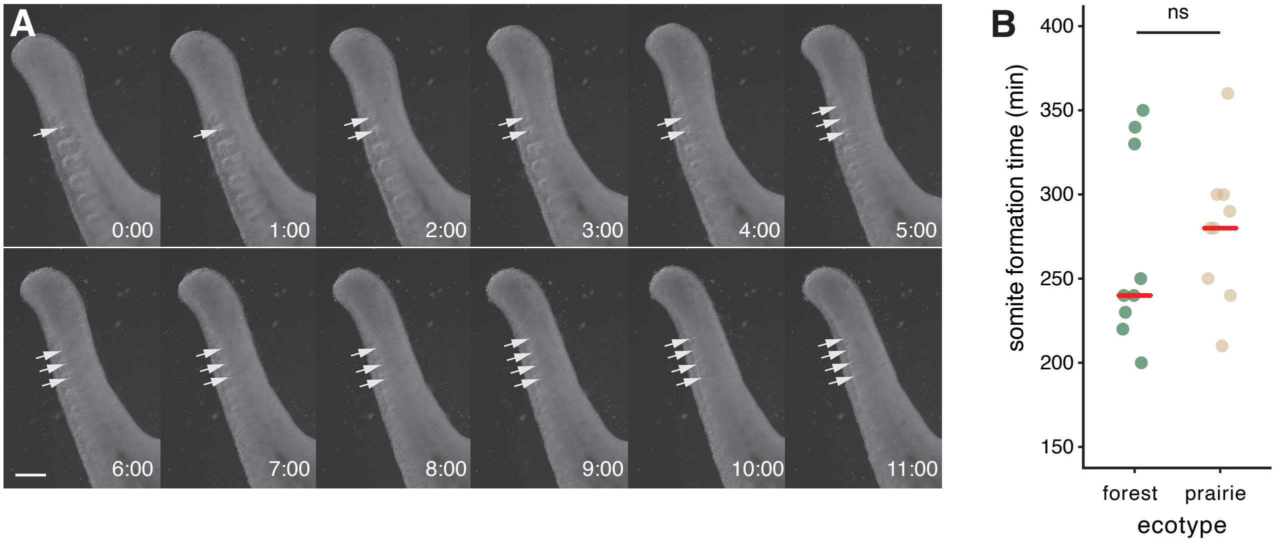

Supplementary Figure 8

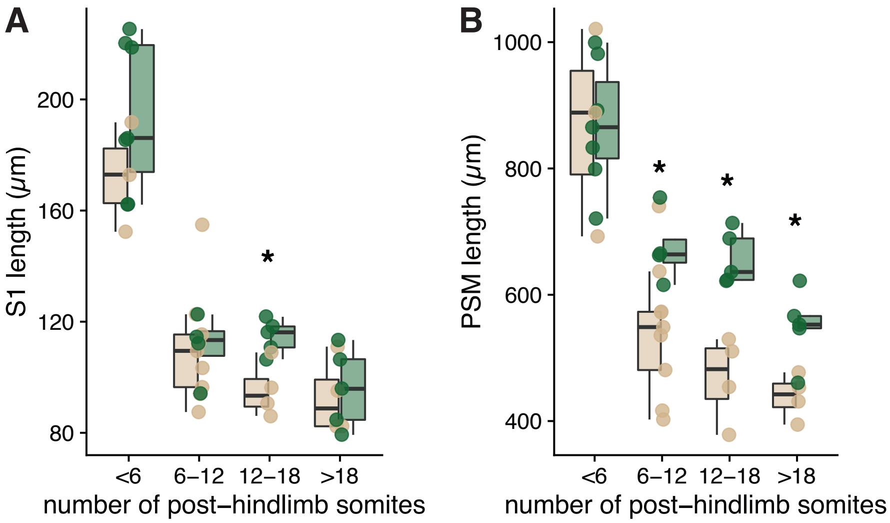

Supplementary Figure 9
