# Supplementary material for: Adaptive tail-length evolution in deer mice is associated with differential Hoxd13 expression in early development
Source: Nat Ecol Evol. 2024 Feb 20;8(4):791–805. doi: 10.1038/s41559-024-02346-3 (PMC11009118; doi:10.1038/s41559-024-02346-3)
Supplement: Supplementary file 2 — Reporting Summary [file 41559_2024_2346_MOESM2_ESM.pdf]

nature portfolio

Corresponding author(s): Evan P Kingsley

Last updated by author(s): Dec 18, 2023

Reporting Summary

Nature Portfolio wishes to improve the reproducibility of the work that we publish. This form provides structure for consistency and transparency in reporting. For further information on Nature Portfolio policies, see our Editorial Policies and the Editorial Policy Checklist.

Statistics

For all statistical analyses, confirm that the following items are present in the figure legend, table legend, main text, or Methods section.

n/a	Confirmed	
☐	☒	The exact sample size (*n*) for each experimental group/condition, given as a discrete number and unit of measurement
☒	☐	A statement on whether measurements were taken from distinct samples or whether the same sample was measured repeatedly
☐	☒	The statistical test(s) used AND whether they are one- or two-sided<br>*Only common tests should be described solely by name; describe more complex techniques in the Methods section.*
☐	☒	A description of all covariates tested
☐	☒	A description of any assumptions or corrections, such as tests of normality and adjustment for multiple comparisons
☐	☒	A full description of the statistical parameters including central tendency (e.g. means) or other basic estimates (e.g. regression coefficient) AND variation (e.g. standard deviation) or associated estimates of uncertainty (e.g. confidence intervals)
☐	☒	For null hypothesis testing, the test statistic (e.g. *F*, *t*, *r*) with confidence intervals, effect sizes, degrees of freedom and *P* value noted<br>*Give P values as exact values whenever suitable.*
☒	☐	For Bayesian analysis, information on the choice of priors and Markov chain Monte Carlo settings
☒	☐	For hierarchical and complex designs, identification of the appropriate level for tests and full reporting of outcomes
☐	☒	Estimates of effect sizes (e.g. Cohen's *d*, Pearson's *r*), indicating how they were calculated

Our web collection on statistics for biologists contains articles on many of the points above.

Software and code

Policy information about availability of computer code

Data collection

Zeiss Zen 2012

Data analysis

R v3.6.2–4.1.1
R packages:
corrplot v0.92
cowplot v1.1.1
edgeR 3.35.3
ggplot2 v3.3.2
ggrepel v0.9.1
Hmisc v4.6.0
limma v3.49.4
lme4 v1.1
psych v2.1.9
qtl v1.50
stats v4.1.1
tximport v1.21.2

Processing ddRAD sequences:
github.com/brantp/rtd
HTseq v0.6.1 in Python v2.7.3

Processing RNAseq sequences:
cutadapt v1.13

RSEM v1.3.0
STAR v2.7.1
Trinity abundance_estimates_to_matrix v2.4.0

For manuscripts utilizing custom algorithms or software that are central to the research but not yet described in published literature, software must be made available to editors and reviewers. We strongly encourage code deposition in a community repository (e.g. GitHub). See the Nature Portfolio guidelines for submitting code & software for further information.

Data

Policy information about availability of data

All manuscripts must include a data availability statement. This statement should provide the following information, where applicable:

- Accession codes, unique identifiers, or web links for publicly available datasets
- A description of any restrictions on data availability
- For clinical datasets or third party data, please ensure that the statement adheres to our policy

The raw and processed forest, prairie, and F1 RNA-seq data have been uploaded to NCBI GEO. Forest and prairie RNAseq data is accession GSE191280 and F1 RNAseq is accession GSE191330. Files containing behavioral assay, QTL mapping, and skeletal morphology data are available on Data Dryad (DOI: 10.5061/dryad.jsxksn0gr).

Field-specific reporting

Please select the one below that is the best fit for your research. If you are not sure, read the appropriate sections before making your selection.

☒ Life sciences ☐ Behavioural & social sciences ☐ Ecological, evolutionary & environmental sciences

For a reference copy of the document with all sections, see nature.com/documents/nr-reporting-summary-flat.pdf

Life sciences study design

All studies must disclose on these points even when the disclosure is negative.

Sample size	We used a power analysis to determine the number of F2 animals in the QTL, such that we could detect additive effects accounting for at least 0.025 of the total F2 variance with alpha = 0.05 and beta = 0.1. Sample sizes of RNAseq, embryo measurement, and cell count experiments were determined by a balance of cost, power, and availability of embryos. We determined a sample size for Mus Hoxd13 CRISPR vertebral counts to produce >70% probability to detect an effect (alpha = 0.05) equivalent to the allelic effect in the deer mouse QTL cross.
Data exclusions	Markers and individuals were filtered according to quality criteria, as described in the methods.
Replication	We used numbers of biological replicates that meet or exceed the standards of the field.
Randomization	Animals were allocated based on their ecotype. We used sacral vertebral length, which does not differ between ecotypes, as a covariate. In the QTL mapping experiment we treated F2s as a single group, so no randomization was required.
Blinding	Investigators did not know genotypes prior to QTL and Hoxd13 CRISPR phenotyping, and were blinded to ecotype/subspecies for embryo measurements and cell counting.

Reporting for specific materials, systems and methods

We require information from authors about some types of materials, experimental systems and methods used in many studies. Here, indicate whether each material, system or method listed is relevant to your study. If you are not sure if a list item applies to your research, read the appropriate section before selecting a response.

Materials & experimental systems

n/a	Involved in the study	
☐	☒	Antibodies
☒	☐	Eukaryotic cell lines
☒	☐	Palaeontology and archaeology
☐	☒	Animals and other organisms
☒	☐	Human research participants
☒	☐	Clinical data
☒	☐	Dual use research of concern

Methods

n/a	Involved in the study	
☒	☐	ChIP-seq
☒	☐	Flow cytometry
☒	☐	MRI-based neuroimaging

Antibodies

Antibodies used

anti-Sox2 (R&D Systems MAB2018; Monoclonal Mouse IgG2A Clone # 245610)
anti-Brachyury/T (R&D Systems AF2085; Polyclonal Goat IgG)

Validation

In our experiments, antibodies label nuclei in the developing neural tube (Sox2) and PSM (T), as expected. Both anti-Sox2 and anti-T antibodies were used in prior studies of NMPs (e.g., for Sox2: López-Escobar, B. et al. The non-canonical Wnt-PCP pathway shapes the mouse caudal neural plate. Development 145, (2018); for T: Guillot, C., Djeffal, Y., Michaut, A., Rabe, B. & Pourquié, O. Dynamics of primitive streak regression controls the fate of neuromesodermal progenitors in the chicken embryo. Elife 10, (2021)).

Animals and other organisms

Policy information about studies involving animals; ARRIVE guidelines recommended for reporting animal research

Laboratory animals

Peromyscus maniculatus (bairdii, nubiterrae subspecies; F1 and F2 hybrids), males and females, age 55–300 days
Mus musculus (C57BL/6J), males and females, P0.

Wild animals

This study did not involve wild animals.

Field-collected samples

This study did not involve animals collected from the field.

Ethics oversight

All breeding colonies and experiments were conducted under and approved by the Harvard IACUC protocol 11-05.

Note that full information on the approval of the study protocol must also be provided in the manuscript.
